# The frequency of cytomegalovirus non-ELR UL146 genotypes in neonates with congenital CMV disease is comparable to strains in the background population

**DOI:** 10.1186/s12879-021-06076-w

**Published:** 2021-04-26

**Authors:** Christian Berg, Mette M. Rosenkilde, Thomas Benfield, Lene Nielsen, Thomas Sundelin, Hans R. Lüttichau

**Affiliations:** 1 Infectious Diseases Unit, Department of Medicine, Copenhagen University Hospital, Herlev Gentofte, DK-2730 Herlev, Denmark; 2 Center for Research and Disruption of Infectious Diseases (CREDID), Department of Infectious Diseases, Copenhagen University Hospital, Amager Hvidovre, DK-2650 Hvidovre, Denmark; 3 Laboratory for Molecular Pharmacology, Department of Biomedical Sciences, Faculty of Health and Medical Sciences, University of Copenhagen, DK-2200 Copenhagen, Denmark; 4 Department of Clinical Microbiology, Copenhagen University Hospital, Herlev Gentofte, DK-2730 Herlev, Denmark

**Keywords:** UL146, vCXCL1, ELR, DBS, cCMV, Cytomegalovirus, Genotyping

## Abstract

**Background:**

Congenital cytomegalovirus disease (cCMV) is common and can be fatal or cause severe sequelae. Circulating strains of cytomegalovirus carry a high number of variable or disrupted genes. One of these is UL146, a highly diverse gene with 14 distinct genotypes encoding a CXC-chemokine involved in viral dissemination. UL146 genotypes 5 and 6 lack the conserved ELR motif, potentially affecting strain virulence. Here, we investigate whether UL146 genotypes 5 and 6 were associated with congenital CMV infection.

**Methods:**

Viral DNA was extracted and UL146 sequenced from 116 neonatal dried blood spots (DBS) stored in the Danish National Biobank since 1982 and linked to registered cCMV cases through a personal identifier. These sequences were compared to UL146 control sequences obtained from CMV DNA extracted from 83 urine samples from children with suspected bacterial urinary tract infections.

**Results:**

Three non-ELR UL146 genotypes (5 and 6) were observed among the cases (2.6%) and two were observed among the controls (2.4%; *P* > 0.99). Additionally, no significant association with cCMV was found for the other 12 genotypes in a post-hoc analysis, although genotype 8 showed a tendency to be more frequent among cases with 12 observations against three (*P* = 0.10). All fourteen genotypes were found to have little intra-genotype variation. Viral load, gender, and sample age were not found to be associated with any particular UL146 genotype.

**Conclusions:**

No particular UL146 genotype was associated with cCMV in this nationwide retrospective case-control study. Associations between CMV disease and disrupted or polymorph CMV genes among immunosuppressed people living with HIV/AIDS and transplant recipients should be investigated in future studies.

**Supplementary Information:**

The online version contains supplementary material available at 10.1186/s12879-021-06076-w.

## Background

Cytomegalovirus (CMV) is a highly prevalent herpesvirus [1] that is commonly transmitted during early childhood [2–4]. Congenital CMV infection (cCMV), the result of mother-to-fetus transmission, is the most common congenital infection in developed countries with an estimated incidence of ~ 0.5% of live births [5, 6]. Ten percent of infected neonates are symptomatic at birth while 15% show sequelae at later stages of their childhood [5, 6]. The symptoms include neurological developmental disorders such as non-hereditary sensorineural hearing loss (SNHL), vision loss, cerebral palsy, intellectual disability, and severe cCMV can lead to pregnancy loss, thus representing a substantial health burden [7].

**Fig. 1:**
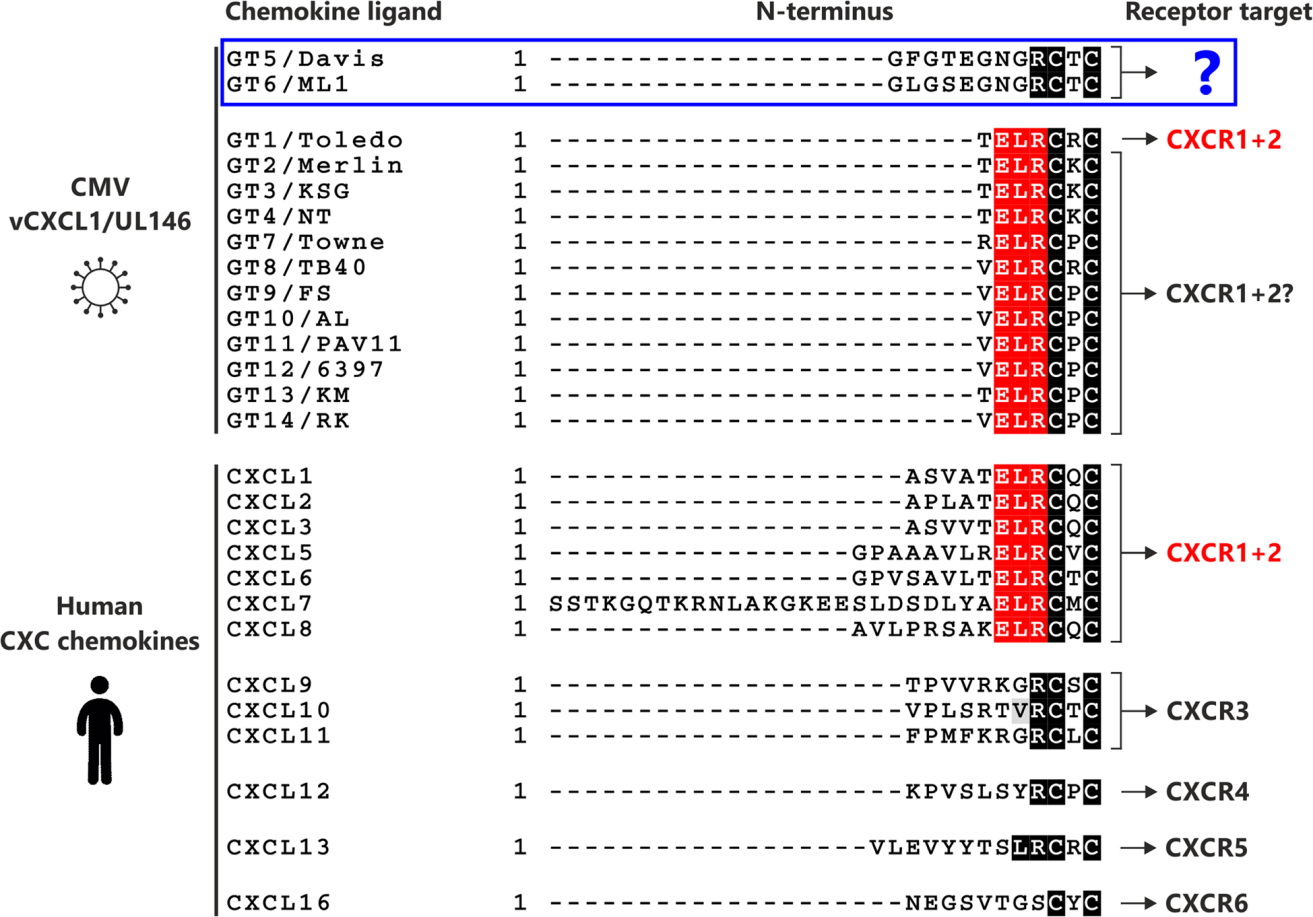
N-terminal amino acid sequence alignment of the different UL146 genotypes and endogenous human CXC chemokines. Genotype 5 and 6 in the blue box have distinct N-termini lacking the ELR motif present in the other 12 UL146 genotypes (names of representative strains encoding each genotype are specified). This motif is conserved in all human CXC chemokines targeting the neutrophil receptors CXCR1 and CXCR2 (CXCL1–8) and lacking in the ones targeting the other CXC chemokine receptors present on different leukocyte subsets (CXCL9–13 and − 16), thus suggesting a different receptor target and biological function of UL146 genotype 5 and 6 (blue question mark). The alignment was generated using ClustalW2

Cytomegalovirus carries a genome of approximately 235 kb double-stranded DNA that encodes numerous genes used in dissemination of infection, immune evasion, and establishment of latent infection. A very high degree of genetic diversity caused by disrupted and highly variable genes has been observed between wild type strains [8, 9]—an unusual observation for DNA viruses—but its effect on the pathogenicity and virulence of individual CMV strains remains to be determined. One of the most diverse genes in the CMV genome is UL146 [8, 9], encoding a viral chemotactic cytokine (chemokine), vCXCL1, which recruits neutrophils to sites of infection through activation of the chemokine receptors CXCR1 and CXCR2 [10, 11]. The first indication that UL146 is a possible virulence gene, and thereby required for CMV infection of the human host, came from the observation that the Toledo wild type strain contained a 15 kb DNA segment containing the UL146 ORF among 22 ORFs not present in laboratory HCMV strains [12]. This indication was recently supported by the finding that recombinant MCMV expressing the UL146 gene enhanced MCMV dissemination kinetics in a mouse [13]. Previous studies have identified 14 distinct UL146 genotypes (GTs) [14] which have shown differences in receptor binding affinity, signaling efficacy, and chemotactic properties [15]. When identifying the UL146 gene product from the Toledo strain (genotype 1) as a CXCR1 and CXCR2 agonist [10], we previously noticed that the sequences of two other genotypes—genotypes 5 and 6—stood out in ways suggesting an altered molecular function likely to have biological implications. Firstly, they lack the ELR motif (amino acids Glu-Leu-Arg) in their N-terminus, a determining factor for chemokine targeting to CXCR1 and CXCR2 (Fig. 1). The ELR motif is conserved in all human chemokines targeting CXCR1 and CXCR2 [16] and is lacking in all chemokines targeting other chemokine receptors. It has been demonstrated, that introduction of an ELR motif in a non-ELR chemokine enables binding to CXCR1 and CXCR2 [17], while a change to any of the three amino acids in an ELR chemokine abolishes its signaling capabilities through CXCR1 and CXCR2 [18]. Secondly, genotypes 5 and 6 have a nine amino acids long N-terminus compared to the only four amino acid of the other UL146 genotypes (C. Berg et al. unpublished observations) (Fig. 1). The N-terminus of a chemokine is crucial for receptor binding and activation as confirmed by the solved structures of chemokine-bound chemokine receptors [19]. Thus, the chemokines encoded by UL146 genotypes 5 and 6 are distinct in ways likely to affect the receptor and host cell target, suggesting an altered biological function of these genotypes.

Previous studies have tried to establish a link between UL146 genotypes and cCMV without finding a clear association [9, 14, 20, 21]. In addition, three studies have investigated whether severe cCMV was linked to specific UL146 genotypes by comparing symptomatic to asymptomatic cCMV cases, but no association was found [22–24]. These studies genotyped UL146 from mostly urine, but also saliva, amniotic fluid, and colon tissue, and were limited by small sample sizes. Another limitation was the lack of control groups representing CMV strains circulating in the background population of healthy individuals. In an attempt to address these problems, we first reasoned the sample size could be considerably increased by using neonatal dried blood spots (DBS) for UL146 genotyping. In many countries, a neonatal DBS is taken at birth for all newborns to screen for select hereditary diseases. These DBS are often stored in biobanks and their use in cCMV diagnosing has been suggested [25–28]. Therefore, we developed a highly efficient extraction and amplification protocol to permit UL146 genotyping from the limited amount of blood available in DBS [29]. Furthermore, we established a control group by screening urine samples from children with a suspected bacterial urine tract infection for CMV and genotyping UL146. Thus, by using the Danish civil registration number individually coupled to diagnoses in the Danish National Patient Registry and neonatal dried blood spots taken from all Danish newborns since 1982 stored in the Danish Neonatal Screening Biobank, we here investigate whether the non-ELR UL146 genotypes affect the development of cCMV disease in 116 infected neonates by comparing to a background population of strains derived from 83 children without CMV disease.

## Methods

### Design, databases and study populations

Free tax-funded healthcare is provided to all residents in Denmark. Every resident is assigned a personal identification number (civil registration number, CPR) and all use of healthcare services is reported to national administrative databases [30]. The Danish National Patient Registry holds information about all hospital admissions and discharge diagnoses [31]. In a nationwide study, cases were identified and CPR-nos. Were extracted from the Danish National Patient Registry on neonates given a diagnosis of congenital CMV disease (diagnosis codes 07950 and DP351) from 1982 to 2018 and a diagnosis of non-hereditary hearing loss caused by CMV disease (diagnosis code DH918A3) from 2010 to 2018 using the International Classification of Diseases 8th and 10th revision. Discharge diagnoses are coded according to the International Classification of Disease (ICD) 8th revision through 1993 and the ICD 10th revision from 1994. The control group was established from urine samples sent to the Department for Microbiology at Herlev-Gentofte Hospital, Denmark, from children aged 1–8 with a suspected bacterial urinary tract infection. It was verified from microbiological databases that the controls were not under investigation for CMV and did not have any previous CMV tests performed. The samples were collected as anonymized excess clinical material.

### Ethics and data protection

The study was approved by the National Committee on Health Research Ethics of the Capital Region of Denmark (record no. H-15017153) and by the Danish Data Protection Agency under the umbrella permit of the Capital Region of Denmark (record no. 2012-58-0004 with local record number VD-2018-101). Civil registration numbers on patients with congenital CMV disease and hearing loss caused by CMV were provided by The Danish Health Data Authority (Sundhedsdatastyrelsen).

### DNA extraction from dried blood spots and urine

For all cases, two DBS punches each 3 mm in diameter were obtained from the Neonatal Screening Biobank in the Danish National Biobank. DNA was extracted as previously described [29], with the two DBS punches being incubated in 1000 μl lysis buffer (600 μl PBS BSA (0.04%), 360 μl Bacterial Lysis Buffer (Roche, Mannheim, Germany) and 40 μl proteinase K (Roche) for 1 h at 55 °C with continuous shaking at low speed. DNA was afterwards extracted from the lysate using a NucliSens easyMAG (bioMérieux) according to manufacturer guidelines using their Specific A protocol with an additional internal lysis step.

For the control group, group, DNA was extracted from 200 μl urine using a NucliSens easyMAG (bioMérieux) according to manufacturer guidelines. The Specific A protocol was used with a final elution volume of 70 μl.

### Quantitative CMV PCR (qPCR)

CMV viral titers (copies/ml) were quantified with the R-gene CMV kit (bioMérieux) according to the manufacturer’s guidelines using a Rotor-gene Q (Qiagen) real-time PCR thermal cycler.

### PCR amplification, purification, and sequencing of UL146

UL146 was PCR amplified, purified, and sequenced as previously described [29] with the exception that PCR of select low CMV titer samples used 10 μl template instead of 5 μl. In details, the PCR mixture contained 1X Pfu DNA polymerase buffer with 2 mM MgSO4, 0.2 mM dNTP (each, Qiagen, Hilden, Germany), 0.5 μM of both forward (CCGGGAATACCGGATATTACG) and reverse primer (CAGCACTTCCTGACGATTG), and 1.25 U Pfu polymerase. The PCR amplification was performed using a ProFlex PCR System (Applied Biosystems, Foster City, USA) thermal cycler. An initial denaturation at 95 °C for 2 min was followed by 40 cycles of denaturation at 95 °C for 30 s, annealing at 61 °C for 30 s, and elongation at 72 °C for 2 min and finally 5 min of final elongation at 72 °C. Samples were stored at 4 °C until analyzed by capillary electrophoresis. After amplification, 10 μl of the PCR product was analyzed on a QIAxcel Advanced System (Qiagen) capillary electrophoresis system using the AM420 screening method, 15 s injection time and one run per row. The QIAxcel DNA Screening kit (Qiagen) was used in combination with the QX Alignment marker 50 bp/5 kb (Qiagen) and QX Size marker 100 bp–2.5 kb (Qiagen). Afterwards, the results were analyzed with the QIAxcel ScreenGel 1.6 software. Samples were purified using the MinElute PCR Purification kit (Qiagen) according to manufacturer instructions before they were Sanger-sequenced by GATC Biotech (Köln, Germany) using the same primers as for PCR amplification (both forward and reverse reads were obtained). Sequence reads were assembled and mapped to a reference sequence in Geneious Prime version 2019.1.3 and genotypes were manually identified. All sequences are available in GenBank under the accession nos. MN929769–MN929883 (cases) and MN929884–MN929966 (controls).

### Statistics

Statistical calculations were performed in RStudio version 1.1 and GraphPad Prism version 7. Fisher’s exact test was performed when comparing genotype observations between the cases and controls. A two-tailed unpaired t-test was implemented to test the difference in viral load between male and female cases and a one-way ANOVA with Tukey’s multiple comparison test between the different genotypes. Pearson’s correlation was used investigate the correlation between and sample age and viral load, and any change was quantified by linear regression analysis.

### Phylogenetic analyses

The phylogenetic analyses were performed in Geneious Prime 2019.1.3. Nucleotide and amino acid sequences were aligned separately using the ClustalW2 algorithm. Phylogenetic trees were generated by Tamura-Nei neighbor-joining of the nucleotide sequence alignment and Jukes-Cantor neighbor-joining of the amino acid sequence alignment. Both trees were resampled by bootstrapping with 1000 replicates and a support threshold of 0%.

### Figures

The chemokine N-terminus alignment and the full sequence alignments were generated with ClustalW (genome.jp/tools-bin/clustalw) and BoxShade version 3.21 (embnet.vital-it.ch/software/BOX_form.html), and the flow chart was created with Lucidchart (lucidchart.com). GraphPad Prism version 7 was used to generate the graphs. Figures were assembled in CorelDraw X6 version 16.

## Results

### UL146 was successfully amplified from 116 out of 120 CMV-positive DBS samples

**Fig. 2:**
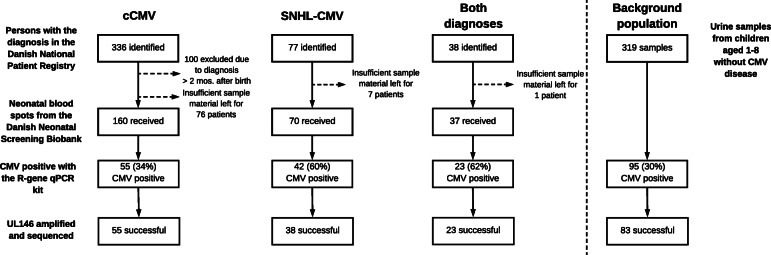
Flow chart of study design and analyzed samples

From the Danish National Patient Registry [31], the personal identification numbers on all Danish patients given the ‘congenital CMV’ (cCMV) diagnosis since 1982 and the ‘innate, non-hereditary hearing loss caused by CMV’ (SNHL-CMV) diagnosis since 2010 were extracted (Fig. 2). Personal identification numbers were obtained on 336 patients in the cCMV group, 77 in the SNHL-CMV group, and 38 who had both diagnoses. For the cCMV group, patients diagnosed more than 2 months after birth were excluded to reduce the risk of including post-natally infected neonates, with the exception of patients presenting with numerous hospital admissions under the cCMV diagnosis, resulting in 236 cCMV patients fulfilling this inclusion criteria. Neonatal blood spots were requested from the Danish Neonatal Screening Biobank for all included patients. As some of the stored blood spots had either been used up over time or only little material reserved for diagnostics remained, the biobank was able to deliver blood spot punches on 160 neonates from the cCMV group, 70 from the SNHL-CMV group, and 37 from the group with both diagnoses. DNA was extracted from the samples and screened for CMV with the R-gene qPCR CMV kit, resulting in 55 CMV positive samples in the cCMV group, 42 in the SNHL-CMV group, and 23 in the group with both diagnoses. Afterwards, UL146 was successfully PCR-amplified in 116 of the total 120 samples (97%). The four failed reactions, all low titer samples belonging to the SNHL-CMV group, were repeated two additional times without success. To test if the UL146 PCR could detect CMV in R-gene negative samples, we randomly selected 20 of these samples for PCR (10 cCMV and 10 SNHL), but all 20 were also found to be negative using the UL146 PCR. To establish a control group, we screened 319 random urine samples from children without a suspected CMV infection aged 1–8 for CMV, resulting in 95 (30%) CMV-positive samples and UL146 was successfully PCR-amplified and sequenced in 83 of them.

### The frequency of non-ELR UL146 genotypes in registered cCMV cases does not differ from the control group

**Fig. 3:**
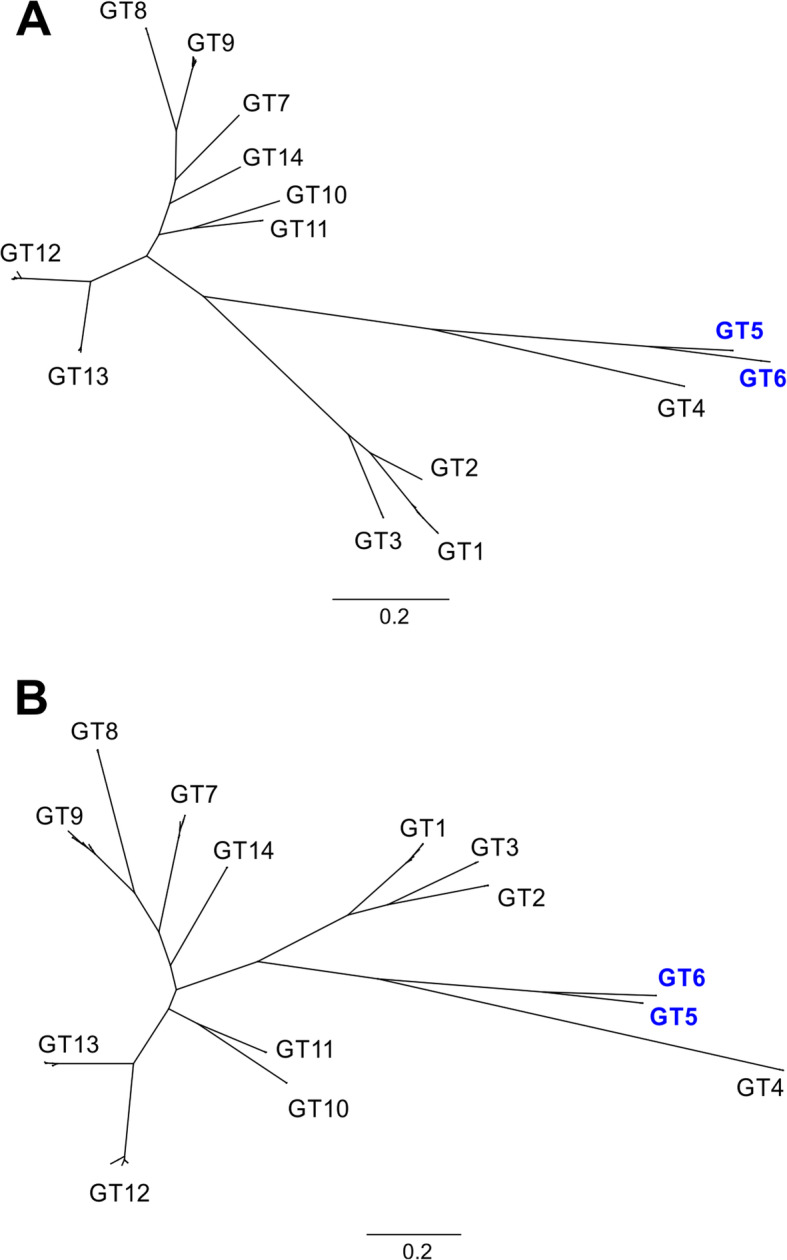
Phylogenetic trees of 198 UL146 nucleotide and amino acid sequences from the cases and controls. **a** Tamura-Nei neighbor-joining of 198 UL146 nucleotide sequences from cases and controls with the 14 previously described genotypes [14]. **b** Jukes-Cantor neighbor-joining of the translated amino acid sequences. One partial genotype 7 sequence was excluded from the tree generation. Trees were resampled by bootstrapping with 1000 replicates. Distance scale presented in substitutions per site

UL146 was sequenced and a phylogenetic analysis of the genotype distribution was performed (Fig. 3). All 199 sequences (116 cases and 83 controls) were found to cluster accordingly with the previously described genotypes [14] for both nucleotide (Fig. 3a) and amino acid sequences (Fig. 3b) and showed very little intra-genotype variation. Among the CMV patients, the overall frequency of non-ELR UL146 genotypes (genotype 5 and 6) was 2.6% (3/116) with all three strains encoding genotype 5 (Table 1), while two (2/83, 2.4%) were observed in the control group. Fisher’s exact test was performed with no significant outcome for genotype 5 and 6 between the groups (*P* > 0.99). Thus, non-ELR genotypes were not found to be associated with CMV disease in neonates.

**Table 1:** Distribution of UL146 genotypes in case and control groups

Genotype	CMV diagnoses						Total case CMV strains		Control CMV strains		***P*** value
	cCMV		SNHL-CMV		cCMV + SNHL-CMV						
	***n***	***f (%)***	***n***	***f (%)***	***n***	***f (%)***	***n***	***f (%)***	***n***	***f (%)***	
**GT1**	2	3.6	3	7.9	0	0.0	**5**	**4.3**	9	10.8	0.09
**GT2**	5	9.1	1	2.6	3	13.0	**9**	**7.8**	3	3.6	0.37
**GT3**	0	0.0	1	2.6	1	4.3	**2**	**1.7**	2	2.4	> 0.99
**GT4**	1	1.8	1	2.6	0	0.0	**2**	**1.7**	2	2.4	> 0.99
**GT5**	1	1.8	1	2.6	1	4.3	**3**	**2.6**	0	0.0	0.27
**GT6**	0	0.0	0	0.0	0	0.0	**0**	**0.0**	2	2.4	0.17
**GT7**	5	9.1	2	5.3	0	0.0	**7**	**6.0**	8	9.6	0.42
**GT8**	7	12.7	2	5.3	3	13.0	**12**	**10.3**	3	3.6	0.10
**GT9**	7	12.7	2	5.3	2	8.7	**11**	**9.5**	8	9.6	> 0.99
**GT10**	1	1.8	2	5.3	0	0.0	**3**	**2.6**	2	2.4	> 0.99
**GT11**	1	1.8	3	7.9	0	0.0	**4**	**3.4**	3	3.6	> 0.99
**GT12**	14	25.5	13	34.2	10	43.5	**37**	**31.9**	26	31.3	> 0.99
**GT13**	9	16.4	6	15.8	3	13.0	**18**	**15.5**	12	14.5	> 0.99
**GT14**	2	3.6	1	2.6	0	0.0	**3**	**2.6**	3	3.6	0.70
**Total**	**55**	**100.0**	**38**	**100.0**	**23**	**100.0**	**116**	**100.0**	**83**	**100.0**	

*P*-value calculated using Fisher’s exact test between total observations among CMV cases compared to the controls

### Analysis of the ELR genotype distribution between cCMV cases and the control group

Among the ELR genotypes in the case group, genotype 12 was the most frequent and observed in 32% of the strains and together with genotype 13 (16%) and genotype 8 (10%) they accounted for 58% of the sequences (Table 1). There was no significant difference in the genotype distribution within the group of congenitally infected neonates or compared to the control group using Fisher’s exact test. The largest discrepancy found between cases and controls were for genotype 1 with 5 cases (4.3%) and 9 controls (10.8%) and genotype 8 with 12 cases (10.3%) and three control (3.6%), with a *P*-values of 0.09 and 0.10, respectively.

### Analysis of intra-genotype variation between strains from cCMV cases and controls

After post hoc analysis for association of specific genotypes with cCMV infection, we investigated whether intra-genotype differences could be associated with cCMV. All amino acid sequences for each UL146 genotype were aligned in order to analyze differences in intra-genotype distributions between strains from cCMV cases and controls of the translated gene (Supplemental data and Table S1). We found two deletions and ten non-conservative mutations appearing more than once in only five strains (Table 2). The frequencies of each mutation/deletion showed no association with cCMV.

**Table 2:** Observed deletions and non-conservative intra-genotype variations among 198 amino acid sequences

Genotype	Intra-genotype variation		Frequencies	
	Non-conservative mutation	Deletion	Case strains	Control strains
GT1		G71-G72	2/4	3/9
	P72H		0/4	2/9
	Y89R		0/4	2/9
	Y89H		2/4	4/9
	G104E		0/4	2/9
	R116G		2/4	3/9
GT9	W41L		4/11	2/8
	W60L		4/11	4/8
	D98G		4/11	3/8
GT11		G100	2/4	0/3
GT12	G55E		13/37	12/26
GT13	G32N		2/19	2/11

Only deletions and non-conservative mutations appearing more than once are shown.

### The CMV-positive rate was higher amongst the SNHL-CMV group than the cCMV group

Overall, 120 of the 267 (45%) blood spots tested positive for CMV, revealing a large portion of the samples from registered CMV cases not containing measurable amounts CMV DNA. The positive rate was different between the two diagnoses at 34% (55/160) for cCMV and 60% (42/70) for SNHL-CMV, which was a highly significant difference (*P* = 0.0005) using Fisher’s exact test.

### Viral titer in cCMV is not associated with UL146 genotype or gender, and not correlated with sample age

**Fig. 4:**
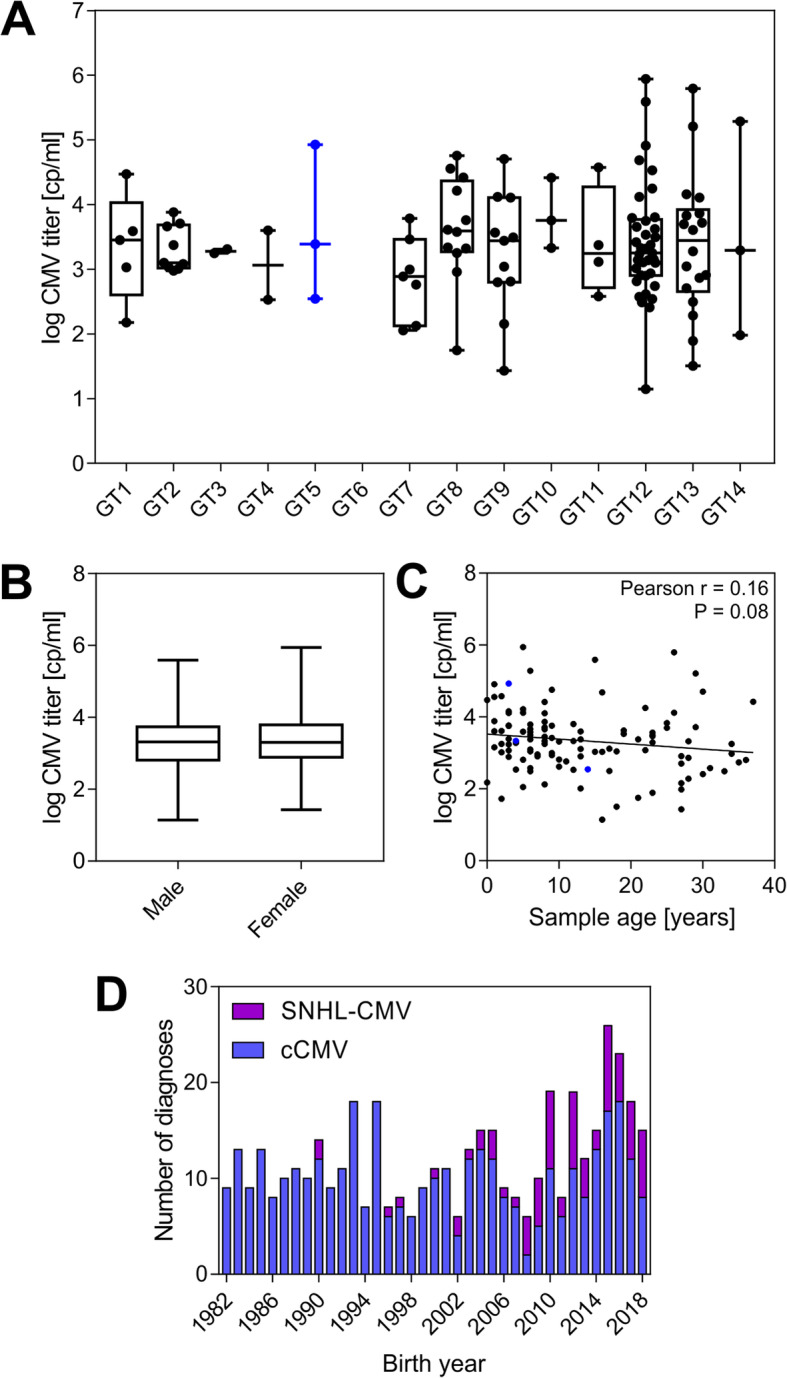
Analysis of viral titer association with UL146 genotype, gender, and sample age. **a** Box-whisker plot of sample CMV titer and the UL146 genotype found in the sample. Non-ELR UL146 genotypes are marked in blue. No significant differences were found in a one-way ANOVA with Tukey’s multiple comparison test, *n* = 116. **b** Box-whisker plot of sample CMV titer and gender showing no association, *n* = 120. **c** Scatter plot of sample age and CMV titer with a linear regression line showing no significant correlation, *n* = 120. Non-ELR UL146 genotypes are marked in blue. **d** Congenital CMV diagnoses over the study period per birth year for a total of 451 registered cases

We did not observe any significant association between CMV titer in the cCMV cases and UL146 genotype in a one-way ANOVA with Tukey’s multiple comparison test (Fig. 4a). We observed an equal number of CMV-positive male and female patients with 60 of each gender and found no significant association between gender and CMV titer in a two-tailed unpaired t-test (*P* = 0.58) after logarithm-transformation of viral titer to achieve a Gaussian distribution (Fig. 4b). Despite expecting the DNA in the DBS samples to degrade over time, sample age was not found to be significantly correlated with viral titer (*P* = 0.079 and *r* = − 0.16[− 0.33, 0.02]) using Pearson’s correlation after logarithm-transformation of age in months and virus titer (Fig. 4c) with linear regression giving a slope of − 0.014 ± 0.008 SE.

## Discussion

Circulating CMV strains exhibit a high degree of genetic diversity unparalleled by other human herpesviruses [8, 9] that affects important immunomodulators. These observations have raised the question of whether specific CMV strains have different pathogenic potential, however, current information has proven insufficient to demonstrate a clear link. UL146 is one of the most diverse CMV genes, encoding a CXC chemokine with 14 distinct genotypes that potentially have different biological functions. Using a control group of circulating CMV strains from the background population, we found no association between the two UL146 genotypes lacking the ELR-motif (genotypes 5 and 6) otherwise conserved in the 12 other genotypes and the development of cCMV disease in neonates.

We genotyped UL146 in a total of 116 CMV positive DBS samples from neonates with cCMV or SNHL-CMV and in 83 samples from children without a history of CMV disease. We observed three genotype 5 strains and no genotype 6 strains in the case group (one in each sub-group of cCMV, SNHL-CMV, and both diagnoses), while two genotype 6 and no genotype 5 strains were observed in the control group, a difference that was not found to be significant. The 2.6% frequency (3/116) of genotype 5 and 6 strains found in neonates in our study did not differ from frequencies found in other studies, which were in the range from 0 to 4.5% [9, 14, 21–24]. This study is yet the largest among UL146 genotyping studies from neonates with cCMV [9, 14, 20–24] and, to our knowledge, the only one with an established control group for comparison of genotype prevalence between patients and the background population. The lack of control groups in previous studies have made it difficult to determine whether the reported prevalence of non-ELR UL146 genotypes in neonates with cCMV is different from the strains circulating in the background population of the particular geographical area. However, there are potential caveats to the comparison of cases and controls. A study sampled longitudinal genomic populations from the urine and plasma of five infants with symptomatic congenital CMV infection. They found that samples from different compartments taken at the same time were more variable than samples from the same compartment taken over time. The data were consistent with models with several bottlenecks and phases of rapid expansion of the viral populations driving viral selection. Furthermore, they found that positive selection played a small role in viral evolution within a compartment in contrast to a strong role and pervasive driver of evolution associated with compartmentalization [32]. Thus, a possible difference in genotypes between cases and controls could also be interpreted as comparing sequences from viral populations drawn from different compartments, i.e. blood and urine, or from comparing sequences from compartments with a high and low viral load. However, no mutations in the UL146 gene between compartments or over time were reported. Likewise, we did not find any inter- or intra-genotypic differences in frequencies of UL146 between the cases and controls (Tables 1 and 2). Though we cannot exclude that genotypes found in children’s urine are not fully representative for strains circulating in the background population, we find it to be a very close approximation as the strains found in childhood remain throughout adult life as CMV establishes latency. Finally, we cannot rule out that non-ELR UL146 genotypes are a pathogenic factor for development of *symptomatic* or *severe* cCMV disease as no medical chart information was obtained, making it impossible to discern the severity of CMV disease between the cases. This study limitation was accepted early on as recent changes in national patient data security regulations required medical chart review to be carried out in local collaborations with doctors from each hospital having treated the patients dating back to 1982, which was not considered feasible. However, as symptomatic cCMV cases are expected to represent a significant part of neonates with a cCMV diagnosis and the prevalence of non-ELR genotypes was low (2.6%), it is not expected that chart review would have changed the conclusions of this report. Post hoc analyses for the other genotypes were performed and the overall genotype distribution was found to be similar between the patient groups and the control population with no significant differences. Furthermore, there was no observable difference in viral load between different UL146 genotypes, which to our knowledge has not been investigated previously. A second limitation is that Sanger sequencing is not suited for detection of multiple UL146 genotypes in mixed CMV infections. However, next generation sequencing has shown that mixed UL146 genotypes are found in just 2% of congenital CMV infections and that multiple CMV strains are infrequently found in congenital infection (14%) [9]. Moreover, NGS performed on enriched target material requires a substantial viral load for full genome coverage. Thus, target enriched HIV samples showed full coverage of the 10 kb HIV genome in 80% of samples with viral load above 3100 cp/ml equivalent to a target input of 1500 viral copies [33]. For comparison, the median viral load in blood in congenital CMV infection has been reported to be 2300 cp/ml [34], equivalent to a DBS target input of approximately 14 copies (from two ~ 3 μl punches), which would suggest a lower sensitivity for UL146 genotyping by NGS than seen for Sanger sequenced PCR products. Thirdly, the use of only one PCR reaction per sample can have allowed for rare polymerase introduced mutations, which could give rise to false positive intra-genotypic mutations. Although the error rate of the *Pfu* polymerase is low, estimated to approx. 1:1000,000 bp, and any minor random error was not likely to affect the manual genotyping of UL146. However for this reason, only deletions and non-conservative mutations appearing more than once are shown in Table 2.

Interestingly, only 451 cases of congenital CMV disease (336 cCMV + 77 SNHL-CMV cases + 38 with both diagnoses) had been registered in Denmark since 1982 with just 267 relevant samples available—much lower than the number expected from reported incidence rates. Assuming a live birth rate of approximately 60.000 per year in Denmark (based on the numbers published by Statistics Denmark) and a cCMV incidence of 0.5% with 10% symptomatic cases, 30 symptomatic cases per year was expected, or 1080 symptomatic cases over the 36 year period, plus a larger number of asymptomatic cases. There was no change in number of diagnosed cCMV cases over this period (Fig. 4d), averaging to 10 cases per year. This suggests that cCMV disease is underreported or underdiagnosed in Denmark.

We were able to detect CMV in 45% of the 267 DBS samples upon screening with the R-gene qPCR CMV kit and found a significantly higher positive rate among samples from neonates with a SNHL-CMV diagnosis compared to those with a cCMV diagnosis. This could be a reflection of the sensitivity of the methods used to extract and detect CMV in the dried blood spots. Sensitivity is a valid concern when using DBS for pathogen detection as only ~ 6 μl dried blood was obtainable from each patient. Despite the extraction protocol being optimized for up to 100% recovery efficiency [29] and the R-gene kit having a high sensitivity (LoD_95%_ of 555 copies/ml and LoD_5%_ of 30 copy/ml), the 6 μl dried blood was diluted during DNA extraction which allowed for cases with very low viral loads to avoid detection. In previous studies, the sensitivity for detecting CMV in DBS from neonates with *confirmed* cCMV by urine/saliva analysis has shown variation depending on the study design. One study found 100% sensitivity for 72 cCMV cases using a relatively large sample volume [35], while another three studies reported sensitivities from 70 to 75% among 55, 70, and 103 cCMV cases [26, 36, 37] which agrees with a report that 75% of cCMV cases are viraemic at birth [38], and a fifth group found a lower sensitivity of 34% for 91 cCMV cases [27] that the authors explain as being related to DNA extraction and PCR. However, all undetectable cases are likely not accounted for by the sensitivity of the assay as there was a significant difference between the subgroups of neonates with a cCMV diagnosis and those with a SNHL-CMV diagnosis in our study. Thus, several factors are likely to influence the positive rate in the case groups. Firstly, the sensitivity of extraction and PCR, as low and non-viraemic cases could be missed. Secondly, the diagnostic precision, as cases could not be validated without chart review. And thirdly, the timing of infection, as the cCMV diagnosis may have been given due to active infection of the mother during pregnancy and not of the child at birth.

## Conclusions

We sequenced UL146 in 116 DBS samples from neonates with cCMV disease, compared the results to 83 samples from the background population, and did not find an association between specific UL146 genotypes and cCMV disease. As studies have shown that circulating CMV strains contain disruptions of more than 25 genes and have at least 30 polymorphic genes [8, 9], each of these individual genes as well as a mix of viral and host factors should be investigated as a whole in future studies looking for an association between genetic content and clinical endpoints.

## Supplementary Information

**Additional file 1.**

**Additional file 2.**

## Data Availability

Patient identifiers were provided by The Danish Health Data Authority and data were extracted from the Danish National Patient Registry following study and data acquisition approval (see relevant permission obtained under “Ethical approval” above). All UL146 sequences generated in the study are available in GenBank (https://www.ncbi.nlm.nih.gov/genbank/) under the accession nos. MN929769–MN929883 (cases, https://www.ncbi.nlm.nih.gov/popset/?term=1928703183) and MN929884–MN929966 (controls, https://www.ncbi.nlm.nih.gov/popset/?term=1928703413). All other datasets used and/or analyzed in the study are available from the corresponding authors upon reasonable request.

## Abbreviations

CMV: Cytomegalovirus
cCMV: Congenital cytomegalovirus disease
SNHL: Sensorineural hearing-loss
ELR: Amino acid Glu-Leu-Arg
DBS: Dried blood spot
CPR: Civil registration number
