# Supplementary material for: The frequency of cytomegalovirus non-ELR UL146 genotypes in neonates with congenital CMV disease is comparable to strains in the background population
Source: BMC Infect Dis. 2021 Apr 26;21:386. doi: 10.1186/s12879-021-06076-w (PMC8077815; doi:10.1186/s12879-021-06076-w)
Supplement: Supplementary file 1 — Additional file 1. [file 12879_2021_6076_MOESM1_ESM.docx]

**Genotypic alignments of vCXCL1 from case strains and control strains:**

- Bold textface are genotypes found in strains from congenital CMV infected neonates.
- Light textface are genotypes found in strains from controls.
- Blue non-conservative amino-acid substitution.
- Yellow conservative amino-acid substitution.
- Green deletion.
- One GT7 sequence was omitted from this analysis due to a non-optimal C-terminal sequence.

**GT1**

**D05a_extraction_1**  MRLIFGALIIFLAYVYHYEVNGTELRCRCLHRKWPPNKIILGNYWLHRDP

**E7a_146F.ab1_extraction_1** MRLIFGALIIFLAYVYHYEVNGTELRCRCLHRKWPPNKIILGNYWLHRDP

0952_146R.ab1__reversed__extra MRLIFGALIIFLAYVYHYEVNGTELRCRCLHRKWPPNKIILGNYWLHRDP

2594_146R.ab1__reversed__extra MRLIFGALIIFLAYVYHYEVNGTELRCRCLHRKWPPNKIILGNYWLHRDP

105631664857_146F.ab1_extracti MRLIFGALIIFLAYVYHYEVNGTELRCRCLHRKWPPNKIILGNYWLHRDP

**C2c_146F.ab1_extraction_1** MRLIFGALIIFLAYVYHYEVNGTELRCRCLHRKWPPNKIILGNYWLHRDP

105601793454#2.ab1__reversed__ MRLIFGALIIFLAYVYHYEVNGTELRCRCLHRKWPPNKIILGNYWLHRDP

105601808028_146R.ab1__reverse MRLIFGALIIFLAYVYHYEVNGTELRCRCLHRKWPPNKIILGNYWLHRDP

B13_146R.ab1__reversed__extrac MRLIFGALIIFLAYVYHYEVNGTELRCRCLHRKWPPNKIILGSYWLHRDP

**H7c_146F.ab1_extraction_1** MRLIFGALIIFLAYVYHYEVNGTELRCRCLHRKWPPNKIILGNYWLHRDP

**C5c_146F.ab1_extraction_1** MRLIFGALIIFLAYVYHYEVNGTELRCRCLHRKWPPNKIILGNYWLHRDP

105600374792_146R.ab1__reverse MRLIFGALIIFLAYVYHYEVNGTELRCRCLHRKWPPNKIILGNYWLHRDP

105632128800#3.ab1__reversed__ MRLIFGALIIFLAYVYHYEVNGTELRCRCLHRKWPPNKIILGNYWLHRDP

105569546756#2.ab1__reversed__ MRLIFGALIIFLAYVYHYEVNGTELRCRCLHRKWPPNKIILGNYWLHRDP

******************************************.*******

**D05a_extraction_1** RGPGCDKNEHLLYPDGRKPPGPGVCLSPDHLFSKWLDKYNDNRWYNVNIT

**E7a_146F.ab1_extraction_1**  RGPGCDKNEHLLYPDGRKPPGPGVCLSPDHLFSKWLDKYNDNRWYNVNIT

0952_146R.ab1__reversed__extra RGPGCDKNEHLLYPDGRKPPGPGVCLSPDHLFSKWLDKYNDNRWYNVNIT

2594_146R.ab1__reversed__extra RGPGCDKNEHLLYPDGRKPPGPGVCLSPDHLFSKWLDKYNDNRWYNVNIT

105631664857_146F.ab1_extracti RGPGCDKNEHLLYPDGRKPPGPGVCLSPDHLFSKWLDKYNDNRWYNVNIT

**C2c_146F.ab1_extraction_1** RGPGCDKNEHLLYPDGRKPPGSGVCLSPDHLFSKWLDKYNDNRWYNVNIT

105601793454#2.ab1__reversed__ RGPGCDKNEHLLYPDGRKPPGHGVCLSPDHLFSKWLDKRNDNRWYNVNIT

105601808028_146R.ab1__reverse RGPGCDKNEHLLYPDGRKPPGHGVCLSPDHLFSKWLDKRNDNRWYNVNIT

B13_146R.ab1__reversed__extrac RGPGCDKNEHLLYPDGRKPPGPGVCLSPDHLFSKWLDKHNDNRWYNVNIM

**H7c_146F.ab1_extraction_1** RGPGCDKNEHLLYPNGKKPP--GVCLSPDHLFSKWLDKHDDNRWYNVNIT

**C5c_146F.ab1_extraction_1** RGPGCDKNEHLLYPNGKKPP--GVCLSPDHLFSKWLDKHDDNRWYNVNIT

105600374792_146R.ab1__reverse RGPGCDKNEHLLYPNGKKPP--GVCLSPDHLFSKWLDKHDDNRWYNVNIT

105632128800#3.ab1__reversed__ RGPGCDKNEHLLYPNGRKPP--GVCLSPDHLFSKWLDKHDDNRWYNVNIT

105569546756#2.ab1__reversed__ RGPGCDKNEHLLYPNGRKPP--GVCLSPDHLFSKWLDKHDDNRWYNVNIT

**************:*:*** **************** :*********

**D05a_extraction_1** KSPGPRRINITLIGVRG

**E7a_146F.ab1_extraction_1** KSPGPRRINITLIGVRG

0952_146R.ab1__reversed__extra KSPGPRRINITLIGVRG

2594_146R.ab1__reversed__extra KSPGPRRINITLIGVRG

105631664857_146F.ab1_extracti KSPGPRRINITLIGVRG

**C2c_146F.ab1_extraction_1** KSPGPRRINITLIGVRG

105601793454#2.ab1__reversed__ KSPEPRRINITLIGVRG

105601808028_146R.ab1__reverse KSPEPRRINITLIGVRG

B13_146R.ab1__reversed__extrac KSPGPRRINITLIGVRG

**H7c_146F.ab1_extraction_1** KSPGPRRINITLIGVGG

**C5c_146F.ab1_extraction_1** KSPGPRRINITLIGVGG

105600374792_146R.ab1__reverse KSPGPRRINITLIGVGG

105632128800#3.ab1__reversed__ KSPGPRRINITLIGVGG

105569546756#2.ab1__reversed__ KSPGPRRINITLIGVGG

*** *********** *

**GT2**

B11_146F.ab1_extraction_1 MRLIFGALIISLTYMYYYEVHGTELRCKCLDGKKLPPKTIMLGNFWFHRE

7994_146R.ab1__reversed__extra MRLIFGALIISLTYMYYYEVHGTELRCKCLDGKKLPPKTIMLGNFWFHRE

5218_146F.ab1_extraction_1 MRLIFGALIISLTYMYYYEVHGTELRCKCLDGKKLPPKTIMLGNFWFHRE

**A7b_146F.ab1_extraction_1** MRLIFGALIISLTYMYYYEVHGTELRCKCLDGKKLPPKTIMLGNFWFHRE

**B6c_146F.ab1_extraction_1** MRLIFGALIISLTYMYYYEVHGTELRCKCLDGKKLPPKTIMLGNFWFHRE

**C8a_146F.ab1_extraction_1** MRLIFGALIISLTYMYYYEVHGTELRCKCLDGKKLPPKTIMLGNFWFHRE

**C11a_146F.ab1_extraction_1** MRLIFGALIISLTYMYYYEVHGTELRCKCLDGKKLPPKTIMLGNFWFHRE

**d2c_extraction_1** MRLIFGALIISLTYMYYYEVHGTELRCKCLDGKKLPPKTIMLGNFWFHRE

**E01a_extraction_1** MRLIFGALIISLTYMYYYEVHGTELRCKCLDGKKLPPKTIMLGNFWFHRE

**G9c_146F.ab1_extraction_1** MRLIFGALIISLTYMYYYEVHGTELRCKCLDGKKLPPKTIMLGNFWFHRE

**F12c_146F.ab1_extraction_1** MRLIFGALIISLTYMYYYEVHGTELRCKCLDGKKLPPKTIMLGNFWFHRE

**C3b_146F.ab1_extraction_1** MRLIFGALIISLTYMYYYEVHGTELRCKCLDGKKLPPKTIMLGNFWFHRE

**************************************************

B11_146F.ab1_extraction_1 SGGPRCNNNEYFLYLGGGKKHGPGVCLSPHHPFSKWLDKRNDNRWYNVNV

7994_146R.ab1__reversed__extra SGGPRCNNNEYFLYLGGGKKHGPGVCLSPHHPFSKWLDKRNDNRWYNVNV

5218_146F.ab1_extraction_1 SGGPRCNNNEYFLYLGGGKKHGPGVCLSPHHPFSKWLDKRNDNRWYNVNV

**A7b_146F.ab1_extraction_1** SGGPRCNNNEYFLYLGGGKKHGPGVCLSPHHPFSKWLDKRNDNRWYNVNV

**B6c_146F.ab1_extraction_1** SGGPRCNNNEYFLYLGGGKKHGPGVCLSPHHPFSKWLDKRNDNRWYNVNV

**C8a_146F.ab1_extraction_1** SGGPRCNNNEYFLYLGGGKKHGPGVCLSPHHPFSKWLDKRNDNRWYNVNV

**C11a_146F.ab1_extraction_1** SGGPRCNNNEYFLYLGGGKKHGPGVCLSPHHPFSKWLDKRNDNRWYNVNV

**d2c_extraction_1** SGGPRCNNNEYFLYLGGGKKHGPGVCLSPHHPFSKWLDKRNDNRWYNVNV

**E01a_extraction_1** SGGPRCNNNEYFLYLGGGKKHGPGVCLSPHHPFSKWLDKRNDNRWYNVNV

**G9c_146F.ab1_extraction_1** SGGPRCNNNEYFLYLGGGKKHGPGVCLSPHHPFSKWLDKRNDNRWYNVNV

**F12c_146F.ab1_extraction_1** SGGPRCNNNEYFLYLGGGKKHGPGVCLSPHHPFSKWLDKRNDNRWYNVNV

**C3b_146F.ab1_extraction_1** SGGPRCNNNEYFLYLGGGKKHGPGVCLSPHHPFSKWLDKRNDNRWYNVNV

**************************************************

B11_146F.ab1_extraction_1 TRQPERGPGKITVTLVGLKE

7994_146R.ab1__reversed__extra TRQPERGPGKITVTLVGLKE

5218_146F.ab1_extraction_1 TRQPERGPGKITVTLVGLKE

**A7b_146F.ab1_extraction_1** TRQPERGPGKITVTLVGLKE

**B6c_146F.ab1_extraction_1** TRQPERGPGKITVTLVGLKE

**C8a_146F.ab1_extraction_1** TRQPERGPGKITVTLVGLKE

**C11a_146F.ab1_extraction_1** TRQPERGPGKITVTLVGLKE

**d2c_extraction_1** TRQPERGPGKITVTLVGLKE

**E01a_extraction_1** TRQPERGPGKITVTLVGLKE

**G9c_146F.ab1_extraction_1** TRQPERGPGKITVTLVGLKE

**F12c_146F.ab1_extraction_1** TRQPERGPGKITVTLVGLKE

**C3b_146F.ab1_extraction_1** TKQPERGPGKITVTLVGLKE

*:******************

**GT3**

105599705356_146F.ab1_extracti MRFIFGLLIIFLAYMYYYEVNGTELRCKCPGDKKLPRTRIMLGDFWTHRE

105600395366_146R.ab1__reverse MRFIFGLLIIFLAYMYYYEVNGTELRCKCPGDKKLPRTRIMLGDFWTHRE

**E11c_146F.ab1_extraction_1** MRFIFGLLIIFLAYMYYYEVNGTELRCKCPGDKKLPRTRIMLGDFWTHRE

**C12b_146F.ab1_extraction_1** MRFIFGLLIIFLAYMYYYEVNGTELRCKCPGDKKLPRTRIMLGDFWTHRE

**************************************************

105599705356_146F.ab1_extracti SGGPGCNGYQYLLYFNNGGKHGRGVCLAPDHHISKWLDTHNDGRWYNVNI

105600395366_146R.ab1__reverse SGGPGCNGYQYLLYFNNGGKHGRGVCLAPDHHISKWLDTHNDGRWYNVNI

**E11c_146F.ab1_extraction_1** SGGPGCNGYQYLLYFNNGGKHGRGVCLAPDHHISKWLDTHNDGRWYNVNI

**C12b_146F.ab1_extraction_1** SGGPGCNGYQYLLYFNNGGKHGRGVCLAPDHHISKWLNTHNDGRWYNVNI

*************************************:************

105599705356_146F.ab1_extracti TKQPGRRTGGRGPGQVNITLIAVKQ

105600395366_146R.ab1__reverse TKQPGRRTGGRGPGQVNITLIAVKQ

**E11c_146F.ab1_extraction_1** TKQPGRRTGGRGPGQVNITLIAVKQ

**C12b_146F.ab1_extraction_1** TKQPGRRTGGRGPGQVNITLIAVKQ

*************************

**GT4**

105600290335_146R.ab1__reverse MRLIFGPFIGLLIACMCYYVWSTELRCKCAGGQSWHPRGKWPTKHHWLEC

B12_146F.ab1_extraction_1 MRLIFGPFIGLLIACMCYYVWSTELRCKCAGGQSWHPRGKWPTKHHWLEC

**B5a_146F.ab1_extraction_1** MRLIFGPFIGLLIACMCYYVWSTELRCKCAGGQSWHPRGKWPTKHHWLEC

**A9c_extraction_1** MRLIFGPFIGLLIACMCYYVWSTELRCKCAGGQSWHPRGKWPTKHHWLEC

**************************************************

105600290335_146R.ab1__reverse YPPSGNCPAGELLIYFEEHNWSPKCVHVHNPFGQKFMSKCDKHEWFEVTF

B12_146F.ab1_extraction_1 YPPSGNCPAGELLIYFEEHNWSPKCVHVHNPFGQKFMSKCDKHEWFEVTF

**B5a_146F.ab1_extraction_1** YPPSGNCPAGELLIYFEEHNWSPKCVHVHNPFGQKFMSKCDKHEWFEVTF

**A9c_extraction_1** YPPSGNCPAGELLIYFEEHNWSPKCVHVHNPFGQKFMSKCDKHEWFEVNF

************************************************.*

105600290335_146R.ab1__reverse NSTRKYPMITRKGSTKPTFSSGK

B12_146F.ab1_extraction_1 NSTRKYPMITRKGSTKPTFSSGK

**B5a_146F.ab1_extraction_1** NSTRKYPMITRKGSTKPTFSSGK

**A9c_extraction_1** NSTRKYPMITRKGSTKPTFSSGK

***********************

**GT5**

**D11c_146F.ab1_extraction_1** MRLIFGLLIIFIVTDTCNGGFGTEGNGRCTCIGYHRLLGQLPRGTFWLGH

**E6c_extraction_1** MRLIFGLLIIFIVTDTCNGGFGTEGNGRCTCIGYHRLLGQLPRGTFWLGH

**F3a_146F.ab1_extraction_1** MRLIFGLLIIFIVTDTCNGGFGTEGNGRCTCIGYHRLLGQLPRGTFWLGH

**************************************************

**D11c_146F.ab1_extraction_1** LPPGSHCPKGQVMIKIGQGPIVCLSDYHPLSKWMYGNHKSGSETWLQIKM

**E6c_extraction_1** LPPGSHCPKGQVMIKIGQGPIVCLSDYHPLSKWMYGNHKSGSETWLQIKM

**F3a_146F.ab1_extraction_1** LPPGSHCPKGQVMIKIGQGPIVCLSDHHPLSKWMYGNHKSGSETWLQIKM

**************************:***********************

**D11c_146F.ab1_extraction_1** EGPRNATVVQRSNTRP

**E6c_extraction_1** EGPRNATVVQRSNTRP

**F3a_146F.ab1_extraction_1** EGPRNATVVQRSNTRP

****************

**GT6**

1083_146F.ab1_extraction_1 MRFIFGLLISLMVAHTCNAGLGSEGNGRCTCVGYHRFDKQLPRGTIWLGH

105633304807_146F.ab1_extracti MRFIFGLLISLMVAHTCNAGLGSEGNGRCTCVGYHRFDKQLPRGTIWLGH

**************************************************

1083_146F.ab1_extraction_1 RPPGPHCPRGDVLMKLGEQPTVCLSDHHPLSKWMYRHHGSDTEIWFQIEF

105633304807_146F.ab1_extracti RPPGPHCPRGDVLMKLGEQPTVCLSDHHPLSKWMYRHHGSDTEIWFQIEF

**************************************************

1083_146F.ab1_extraction_1 KGPQNTKVVSKSFTPPS

105633304807_146F.ab1_extracti KGPQNTKVVSKSFTPPS

*****************

**GT7**

6797_146R.ab1__reversed__extra MRLIFGSLISLLMAFMYYHGVHSRELRCPCTHKALHHPIGGLFWVGRDPP

9621_146F.ab1_extraction_1 MRLIFGSLISLLMAFMYYHGVHSRELRCPCTHKALHHPIGGLFWVGRDPP

105601896008#4.ab1__reversed__ MRLIFGSLISLLMAFMYYHGVHSRELRCPCTHKALHHPIGGLFWVGRDPP

105633443432_146R.ab1__reverse MRLIFGSLISLLMAFMYYHGVHSRELRCPCTHKALHHPIGGLFWVGRDPP

1536_146R.ab1__reversed__extra MRLIFGSLISLLMAFMYYHGVHSRELRCPCTHKALHHPIGGLFWVGRDPP

**F7b_146F.ab1_extraction_1** MRLIFGSLISLLMAFMYYHGVHSRELRCPCTHKALHHPIGGLFWVGRDPP

5193_146R.ab1__reversed__extra MRLIFGSLISLLMAFMYYHGVHSRELRCPCTHKALHHPIGGLFWVARDPP

**A12c_146F.ab1_extraction_1** MRLIFGSLISLLMAFMYYHGVHSRELRCPCTHKALHHPIGGLFWVGRDPP

**E3a_146F.ab1_extraction_1** MRLIFGSLISLLMAFMYYHGVHSRELRCPCTHKALHHPIGGLFWVGRDPP

**E12c_extraction_1** MRLIFGSLISLLMAFMYYHGVHSRELRCPCTHKALHHPIGGLFWVGRDPP

**F9a_146F.ab1_extraction_1** MRLIFGTLISLLMAFMYYHGVHSRELRCPCTHKALHHPIGGLFWVGRDPP

2960_146R.ab1__reversed__extra MRLIFGSLISLLMAFMYYHGVHSRELRCPCTHKALHHPIGGLFWVGRDPP

105600283061_146F.ab1_extracti MRLIFGSLISLLMAFMYYHGVHSRELRCPCTHKALHHPIGGLFWVGRDPP

**F5a_146F.ab1_extraction_1** MRLIFGSLISLLMAFMYYHGVHSRELRCPCTHKALHHPIGGLFWVGRDPP

******:**************************************.****

6797_146R.ab1__reversed__extra NPPECDKPQHYLLPPRGKPVCLAPDHHLSKWLDGKKDNSWHKVLVKVKDS

9621_146F.ab1_extraction_1 NPPECDKPQHYLLPPRGKPVCLAPDHHLSKWLDGKKDNSWHKVLVKVKDS

105601896008#4.ab1__reversed__ NPPECDKPQHYLLPPRGKPVCLAPDHHLSKWLDGKKDNSWHKVLVKVKDS

105633443432_146R.ab1__reverse NPPECDKPQHYLLPPRGKPVCLAPDHHLSKWLDGKKDNSWHKVLVKVKDS

1536_146R.ab1__reversed__extra NPPECDKPQHYLLPPRGKPVCLAPDHHLSKWLDGKKDNSWHKVLVKVKDS

**F7b_146F.ab1_extraction_1** NPPECDKPQHYLLPPRGKPVCLAPDHHLSKWLDGKKDNSWHKVLVKVKDS

5193_146R.ab1__reversed__extra NPPECDKPQHYLLPPRGKPVCLAPDHHLSKWLDGKKDNSWHKVLVKVKDS

**A12c_146F.ab1_extraction_1** NPPECDKPQHYLLPPRGKPVCLAPDHHLSKWLDGKKDNSWHKVLVKVKDS

**E3a_146F.ab1_extraction_1** NPPECDKPQHYLLPPRGKPVCLAPDHHLSKWLDGKKDNSWHKVLVKVKDS

**E12c_extraction_1** NPPECDKPQHYLLPPRGKPVCLAPDHHLSKWLDGKKDNSWHRVLVRVKDS

**F9a_146F.ab1_extraction_1** NPPECDKPQHYLLPPRGKPVCLAPDHHLSKWLDGKKDNSWHRVLVKVKDS

2960_146R.ab1__reversed__extra NPPECDKPQHYLLPPRGKPVCLAPDHHLSKWLDGKKDNSWHRVFVKVKDS

105600283061_146F.ab1_extracti NPPECDKPQHYLLPPRGKPVCLAPDHHLSKWLDGKKDNSWHRVFVKVKDS

**F5a_146F.ab1_extraction_1** NPPECDKPQHYLLPPRGKPVCLAPDHHLSKWLDGKKDNSWHKVLVKVKDS

*****************************************:*:*:****

6797_146R.ab1__reversed__extra NGPHVEENAVTNKRPRWK

9621_146F.ab1_extraction_1 NGPHVEENAVTNKRPRWK

105601896008#4.ab1__reversed__ NGPHVEENAVTNKRPRWK

105633443432_146R.ab1__reverse NGPHVEENAVTNKRPRWK

1536_146R.ab1__reversed__extra NGPHVEENAVTNKRPRWK

**F7b_146F.ab1_extraction_1** NGPHVEENAVTNKRPRWK

5193_146R.ab1__reversed__extra NGPHVEENAVTNKRPRWK

**A12c_146F.ab1_extraction_1** NGPHVEENAVTNKRPRWK

**E3a_146F.ab1_extraction_1** NGPHVEENAVTNKRPRWK

**E12c_extraction_1** NGPHVEENAVTNKRPRWK

**F9a_146F.ab1_extraction_1** NGPHVEENAVTNKRPRWK

2960_146R.ab1__reversed__extra NGPHVEENAVTNKRPRWK

105600283061_146F.ab1_extracti NGPHVEENAVTNKRPRWK

**F5a_146F.ab1_extraction_1** NEPHVQENAVTNKRPRWK

* ***:************

**GT8**

B10_146F.ab1_extraction_1 MRFIFSLFGLLIALCYKVESVELRCRCSNGSNHPVFGVFWVGYKPPDPTC

105599705437_146R.ab1__reverse MRFIFSLFGLLIALCYKVESVELRCRCSNGSNHPVFGVFWVGYKPPDPTC

105571030362#2.ab1__reversed__ MRFIFSLFGLLIALCYKVESVELRCRCSNGSNHPVFGVFWVGYKPPDPTC

**B11b_146F.ab1_extraction_1** MRFIFSLFGLLIALCYKVESVELRCRCSNGSNHPVFGVFWVGYKPPDPTC

**F9c_146F.ab1_extraction_1** MRFIFSLFGLLIALCYKVESVELRCRCSNGSNHPVFGVFWVGYKPPDPTC

**F10a_146F.ab1_extraction_1** MRFIFSLFGLLIALCYKVESVELRCRCSNGSNHPVFGVFWVGYKPPDPTC

**E7c_146F.ab1_extraction_1** MRFIFSLFGLLIALCYKVESVELRCRCSNGSNHPVFGVFWVGYKPPDPTC

**E2a_146F.ab1_extraction_1** MRFIFSLFGLLIALCYKVESVELRCRCSNGSNHPVFGVFWVGYKPPDPTC

**D12a_146F.ab1_extraction_1** MRFIFSLFGLLIALCYKVESVELRCRCSNGSNHPVFGVFWVGYKPPDPTC

**D10a_146F.ab1_extraction_1** MRFIFSLFGLLIALCYKVESVELRCRCSNGSNHPVFGVFWVGYKPPDPTC

**D7c_146F.ab1_extraction_1** MRFIFSLFGLLIALCYKVESVELRCRCSNGSNHPVFGVFWVGYKPPDPTC

**C5a_146F.ab1_extraction_1** MRFIFSLFGLLIALCYKVESVELRCRCSNGSNHPVFGVFWVGYKPPDPTC

**A4a_146F.ab1_extraction_1** MRFIFSLFGLLIALCYKVESVELRCRCSNGSNHPVFGVFWVGYKPPDPTC

**A2c_146F.ab1_extraction_1** MRFIFSLFGLLIALCYKVESVELRCRCSNGSNHPVFGVFWVGYKPPDPTC

**C10b_146F.ab1_extraction_1** MRFIFSLFGLLIALCYKVESVELRCRCSNGSNHPVFGVFWVGYKPPDPTC

**************************************************

B10_146F.ab1_extraction_1 DKTQHFLLPPRQTPVCLSPDHYLSKWVDGKRSNWWHKVFIKKNSDNGPHI

105599705437_146R.ab1__reverse DKTQHFLLPPRQTPVCLSPDHYLSKWVDGKRSNWWHKVFIKKNSDNGPHI

105571030362#2.ab1__reversed__ DKTQHFLLPPRQTPVCLSPDHYLSKWVDGKRSNWWHKVFIKKNSDNGPHI

**B11b_146F.ab1_extraction_1**  DKTQHFLLPPRQTPVCLSPDHYLSKWVDGKRSNWWHKVFIKKNSDNGPHI

**F9c_146F.ab1_extraction_1** DKTQHFLLPPRQTPVCLSPDHYLSKWVDGKRSNWWHKVFIKKNSDNGPHI

**F10a_146F.ab1_extraction_1** DKTQHFLLPPRQTPVCLSPDHYLSKWVDGKRSNWWHKVFIKKNSDNGPHI

**E7c_146F.ab1_extraction_1** DKTQHFLLPPRQTPVCLSPDHYLSKWVDGKRSNWWHKVFIKKNSDNGPHI

**E2a_146F.ab1_extraction_1** DKTQHFLLPPRQTPVCLSPDHYLSKWVDGKRSNWWHKVFIKKNSDNGPHI

**D12a_146F.ab1_extraction_1** DKTQHFLLPPRQTPVCLSPDHYLSKWVDGKRSNWWHKVFIKKNSDNGPHI

**D10a_146F.ab1_extraction_1** DKTQHFLLPPRQTPVCLSPDHYLSKWVDGKRSNWWHKVFIKKNSDNGPHI

**D7c_146F.ab1_extraction_1** DKTQHFLLPPRQTPVCLSPDHYLSKWVDGKRSNWWHKVFIKKNSDNGPHI

**C5a_146F.ab1_extraction_1** DKTQHFLLPPRQTPVCLSPDHYLSKWVDGKRSNWWHKVFIKKNSDNGPHI

**A4a_146F.ab1_extraction_1** DKTQHFLLPPRQTPVCLSPDHYLSKWVDGKRSNWWHKVFIKKNSDNGPHI

**A2c_146F.ab1_extraction_1** DKTQHFLLPPRQTPVCLSPDHYLSKWVDGKRSNWWHKVFIKKNSDNGPHI

**C10b_146F.ab1_extraction_1** DKTQHFLLPPRQTPVCLSPDHYLSKWVDGKRSNWWHKVFIKKNTDNGPHI

*******************************************:******

B10_146F.ab1_extraction_1 EDKSDTNRHPPWRL

105599705437_146R.ab1__reverse EDKSDTNRHPPWRL

105571030362#2.ab1__reversed__ EDKSDTNRHPPWRL

**B11b_146F.ab1_extraction_1** EDKSDTNRHPPWRL

**F9c_146F.ab1_extraction_1** EDKSDTNRHPPWRL

**F10a_146F.ab1_extraction_1** EDKSDTNRHPPWRL

**E7c_146F.ab1_extraction_1** EDKSDTNRHPPWRL

**E2a_146F.ab1_extraction_1** EDKSDTNRHPPWRL

**D12a_146F.ab1_extraction_1** EDKSDTNRHPPWRL

**D10a_146F.ab1_extraction_1** EDKSDTNRHPPWRL

**D7c_146F.ab1_extraction_1** EDKSDTNRHPPWRL

**C5a_146F.ab1_extraction_1** EDKSDTNRHPPWRL

**A4a_146F.ab1_extraction_1** EDKSDTNRHPPWRL

**A2c_146F.ab1_extraction_1** EDKSDTNRHPPWRL

**C10b_146F.ab1_extraction_1** EDKSDTNRHPPWRL

**************

**GT9**

**A2b_146F.ab1_extraction_1** MRFIFSLFGLLIVLYYKVESMELRCPCGSNGLSYPIGGFFWIGYNPPDPP

**H11a_146F.ab1_extraction_1** MRFIFSLFGLLIVLYYKVESMELRCPCGSNGLSYPIGGFFWIGYNPPDPP

105600783684_146F.ab1_extracti MRFIFSLFGLLIVLYYKVESMELRCPCGSNGLSYPIGGFFWIGYNPPDPP

105632659210_146F.ab1_extracti MRFIFSLFGLLIVLYYKVESMELRCPCGSNGLSYPIGGFFWIGYNPPDPP

**F11a_146F.ab1_extraction_1** MRFIFSLFGLLIVLYYKVESMELRCPCGSNGLSYPIGGLFLIGYNPPDPP

**G8a_146F.ab1_extraction_1** MRFIFSLFGLLIVLYYKVESMELRCPCGSNGLSYPIGGLFLIGYNPPDPP

B14_146R.ab1__reversed__extrac MRFIFSLFGLLIVLYYKVESMELRCPCGSNGLSYPIGGLFLIGYNPPDPP

5318_146F.ab1_extraction_1 MRFIFSLFGLLIVLYYKVESMELRCPCGSNGLSYPIGGLFLIGYNPPDPP

**B9c_extraction_1** MRFIFSLFGLLIALYYKVESMELRCPCGSNGLSYPIGGLFLIGYNPPDPP

**E4c_146F.ab1_extraction_1** MRFIFSLFGLLIVLYYKVESMELRCPCGSNGLSYPIGGLFLIGYNPPDPP

3826_146R.ab1__reversed__extra MRFIFSLFGLLIALYYKVESVELRCPCGSNGLSYPIGGFFWIGYNPPDPP

599_146R.ab1__reversed__extrac MRFIFSLFGLLIALYYKVESVELRCPCGSNGLSYPIGGFFWIGYNPPDPP

**B3b_146F.ab1_extraction_1** MRFIFSLFGLLIALYYKVESVELRCPCGSNGLSYPIGGFFWIGYNPPDPP

2012_146R.ab1__reversed__extra MRFIFSLFGLLIALYYKVESVELRCPCGSNGLSYPIGGFFWIGYNPPDPP

**F4c_146F.ab1_extraction_1** MRFIFSLFGLLIALYYKVESVELRCPCGSNGLSYPIGGFFWIGYNPPDPP

**C1b_146F.ab1_extraction_1** MRFIFSLFGLLIALYYKVESVELRCPCGSNGLSYPIGGFFWIGYNPPDPP

**B5b_146F.ab1_extraction_1** MRFIFSLFGLLIALYYKVESVELRCPCGSNGLSYPIGGFFWIGYNPPDPP

2610_146R.ab1__reversed__extra MRFIFSLFGLLIALYYKVESVELRCPCGSNGLSLPIGGFFWIGYNPPDPP

**H1c_146F.ab1_extraction_1** MRFIFSLFGLLIALYYKVESVELRCPCGSNGLSYPIGGFFWVGYNPPDPP

************.*******:************ ****:* :********

**A2b_146F.ab1_extraction_1**  KCEKPQHFLWPPKGKPVCLSPDHVLSKWLHGKSSNTWHKVLLKTKGGDGP

**H11a_146F.ab1_extraction_1** KCEKPQHFLWPPKGKPVCLSPDHVLSKWLHGKSSNTWHKVLLKTKGGDGP

105600783684_146F.ab1_extracti KCEKPQHFLWPPKGKPVCLSPDHVLSKWLHGKSSNTWHKVLLKTKGGDGP

105632659210_146F.ab1_extracti KCEKPQHFLWPPKGKPVCLSPDHVLSKWLHGKSSNTWHKVLLKTKGGDGP

**F11a_146F.ab1_extraction_1** KCEKPQHFLWPPKGKPVCLSPDHVLSKWLHGKSSNTWHKVLLRTKGGDGP

**G8a_146F.ab1_extraction_1** KCEKPQHFLWPPKGKPVCLSPDHVLSKWLHGKSSNTWHKVLLRTKGGDGP

B14_146R.ab1__reversed__extrac KCEKPQHFLWPPKGKPVCLSPDHVLSKWLHGKSSNTWHKVLLRTKGGDGP

5318_146F.ab1_extraction_1 KCEKPQHFLWPPKGKPVCLSPDHVLSKWLHGKSSNTWHKVLLRTKGGDGP

**B9c_extraction_1** KCEKPQHFLWPPKGKPVCLSPDHVLSKWLHGKSSNTWHKVLLRTKGGDGP

**E4c_146F.ab1_extraction_1** KCEKPQHFLWPPKGKPVCLSPDHVLSKWLHGKSSNTWHKVLLRTKGGDGP

3826_146R.ab1__reversed__extra KCEKPQHFLLPPKGKPVCLSPDHVLSKWLHGKSSNTWHKVLLRTKGGGGP

599_146R.ab1__reversed__extrac KCEKPQHFLLPPKGKPVCLSPDHVLSKWLHGKSSNTWHKVLLRTKGGGGP

**B3b_146F.ab1_extraction_1** KCEKPQHFLLPPKGKPVCLSPDHVLSKWLHGKSSNTWHKVLLRTKGGGGP

2012_146R.ab1__reversed__extra KCEKPQHFLLPPKGKPVCLSPDHVLSKWLHGKSSNTWHKVLLRTKGGGGP

**F4c_146F.ab1_extraction_1** KCEKPQHFLLPPKGKPVCLSPDHVLSKWLHGKSSNTWHKVLLRTKGGGGP

**C1b_146F.ab1_extraction_1** KCEKPQHFLLPPKGKPVCLSPDHVLSKWLHGKSSNTWHKVLLRTKGGGGP

**B5b_146F.ab1_extraction_1** KCEKPQHFLLPPKGKPVCLSPDHVLSKWLHGKSSNTWHKVLLRTKGGGGP

2610_146R.ab1__reversed__extra KCEKPQHFLLPPKGKPVCLSPDHVLSKWLHGKSSNTWHKVLLRTKGGDGP

**H1c_146F.ab1_extraction_1** KCEKPQHFLWPLKGKPVCLSPDHVLSKWLHGKLSNTWHKVLLRTKGGDEP

********* * ******************** *********:****. *

**A2b_146F.ab1_extraction_1** HVEERTASNGRPPWKLKF

**H11a_146F.ab1_extraction_1** HVEERTASNGRPPWKLKF

105600783684_146F.ab1_extracti HVEERTASNGRPPWKLKF

105632659210_146F.ab1_extracti HVEERTASNGRPPWKLKF

**F11a_146F.ab1_extraction_1** HVEEKTASNGRPPWKLKF

**G8a_146F.ab1_extraction_1** HVEEKTASNGRPPWKLKF

B14_146R.ab1__reversed__extrac HVEEKTASNGRPPWKLKF

5318_146F.ab1_extraction_1 HVEEKTASNGRPPWKLKF

**B9c_extraction_1** HVEEKTASNGRPPWKLKF

**E4c_146F.ab1_extraction_1** HVEERTASNGRPPWKLKF

3826_146R.ab1__reversed__extra HVEERTASNGRPPWKLKF

599_146R.ab1__reversed__extrac HVEERTASNGRPPWKLKF

**B3b_146F.ab1_extraction_1** HVEERTASNGRPPWKLKF

2012_146R.ab1__reversed__extra HVEERTASNGRPPWKLKF

**F4c_146F.ab1_extraction_1** HVEERTASNGRPPWKLKF

**C1b_146F.ab1_extraction_1** HVEERTASNGRPPWKLKF

**B5b_146F.ab1_extraction_1** HVEERTASNGRPPWKLKF

2610_146R.ab1__reversed__extra HVEERTASNGRPPWKLKF

**H1c_146F.ab1_extraction_1** HVEEKTASNGRPPWKLKF

****:*************

**GT10**

**A10c_146F.ab1_extraction_1** MRLIFGPLISILIVCYYGVESVELRCPCGSNSVNKPVSGVFLIGRDPPNP

**H4c_146F.ab1_extraction_1** MRLIFGPLISILIVCYYGVESVELRCPCGSNSVNKPVSGVFLIGRDPPNP

**C12a_146F.ab1_extraction_1** MRLIFGPLISILIVCYYGVESVELRCPCGSNSVNKPVSGVFLIGRDPPNP

105600538728_146R.ab1__reverse MRLIFGPLISILIVCYYGVESVELRCPCGSNSVNKPVSGVFLIGRDPPNP

6614_146R.ab1__reversed__extra MRLIFGPLISILIVCYYGVESVELRCPCGSNSVNKPVSGVFLIGRDPPNP

**************************************************

**A10c_146F.ab1_extraction_1** PGCNRFQYYLAPPHGKPVCLDSEHHISKWLDGQNSNSWYKVIIKNGDDNK

**H4c_146F.ab1_extraction_1** PGCNRFQYYLAPPHGKPVCLDSEHHISKWLDGQNSNSWYKVIIKNGDDNK

**C12a_146F.ab1_extraction_1** PGCNRFQYYLAPPHGKPVCLDSEHHISKWLDGQNSNSWYKVIIKNGDDNK

105600538728_146R.ab1__reverse PGCNRFQYYLAPPHGKPVCLDSEHHISKWLDGQNSNSWYKVIIKNGDDNK

6614_146R.ab1__reversed__extra PGCNRFQYYLAPPHGKPVCLDSEHHISKWLDGQNSNSWYKVIIKNGDDNK

**************************************************

**A10c_146F.ab1_extraction_1** PKVEKRTEIKKRFKWN

**H4c_146F.ab1_extraction_1** PKVEKRTEIKKRFKWN

**C12a_146F.ab1_extraction_1** PKVEKRTEIKKRFKWN

105600538728_146R.ab1__reverse PKVEKRTEIKKRFKWN

6614_146R.ab1__reversed__extra PKVEKRTEIKKRFKWN

****************

**GT11**

105633318778_146R.ab1__reverse MRLIFGSLISILIVCYYGVESVELRCPCGSNGLRNPLSGVFLVGRDPPRP

105601808052#2.ab1__reversed__ MRLIFGSLISILIVCYYGVESVELRCPCGSNGLRNPLSGVFLVGRDPPRP

**D12b_146F.ab1_extraction_1** MRLIFGSLISILIVCYYGVESVELRCPCGSNGLRNPLSGVFLVGRDPPRP

3900_146R.ab1__reversed__extra MRLIFGSLISILIVCYYGVESVELRCPCGSNGLRNPLSGVFLVGRDPPRP

**H8c_146F.ab1_extraction_1** MRLIFGSLISILIVCYYGVESVELRCPCGSNGLRNPLSGVFLVGRDPPRP

**F7c_extraction_1** MRLIFGSLISILIVCYYGVESVELRCPCGSNGLRNPLSGVFLVGRDPPRP

**B10c_extraction__reversed__1** MRLIFGSLISILIVCYYGVESVELRCPCGSNGLRNPLSGVFLVGRDPPRP

**************************************************

105633318778_146R.ab1__reverse PGCTKHQHYLVPPNGRRACLNPDHHLSKWLDAQNSNSWYKVVVTTGGGGG

105601808052#2.ab1__reversed__ PGCTKHQHYLVPPNGRRACLNPDHHLSKWLDAQNSNSWYKVVVTTGGGGG

**D12b_146F.ab1_extraction_1** PGCTKHQHYLVPPNGRRACLNPDHHLSKWLDAQNSNSWYKVVVTTGGGGG

3900_146R.ab1__reversed__extra PGCTKHQHYLVPPNGRRACLNPDHHLSKWLDAQNSNSWYKVVVTTGGGGG

**H8c_146F.ab1_extraction_1** PGCTKHQHYLVPPNGRRACLNPDHHLSKWLDAQNSNSWYKVVVTTGGGGG

**F7c_extraction_1** PGCTKHQHYLVPPNGRRACLNPDHHLSKWLDAQNSNSWYKVVVTTGGGG-

**B10c_extraction__reversed__1** PGCTKHQHYLVPPNGRRACLNPDHHLSKWLDAQNSNSWYKVVVTTGGGG-

*************************************************

105633318778_146R.ab1__reverse PHVDKKAEIKKRLS

105601808052#2.ab1__reversed__ PHVDKKAEIKKRLS

**D12b_146F.ab1_extraction_1** PHVDKKAEIKKRLS

3900_146R.ab1__reversed__extra PHVDKKAEIKKRLS

**H8c_146F.ab1_extraction_1** PHVDKKAEIKKRLS

**F7c_extraction_1** PHVDKKAEIKKRLS

**B10c_extraction__reversed__1** PHVDKKAEIKKRLS

**************

**GT12**

**A6c_146F.ab1_extraction_1** MRFIFGLLVSLVIVYTYYYEVQSVELRCPCTDGFRNPLFGLFLIGHDPPH

**D03a_extraction__reversed__1** MRFIFGLLVSLVIVYTYYYEVQSVELRCPCTDGFRNPLFGLFLIGHDPPH

**C1a_146F.ab1_extraction_1** MRFIFGLLVSLVIVYTYYYEVQSVELRCPCTDGFRNPLFGLFLIGHDPPH

**A10a_extraction__reversed__1** MRFIFGLLVSLVIVYTYYYEVQSVELRCPCTDGFRNPLFGLFLIGHDPPH

**D11b_146F.ab1_extraction_1** MRFIFGLLVSLVIVYTYYYEVQSVELRCPCTDGFRNPLFGLFLIGHDPPH

**E11b_146F.ab1_extraction_1** MRFIFGLLVSLVIVYTYYYEVQSVELRCPCTDGFRNPLFGLFLIGHDPPH

**F5b_146F.ab1_extraction_1** MRFIFGLLVSLVIVYTYYYEVQSVELRCPCTDGFRNPLFGLFLIGHDPPH

**F6a_146F.ab1_extraction_1** MRFIFGLLVSLVIVYTYYYEVQSVELRCPCTDGFRNPLFGLFLIGHDPPH

**A5c_146F.ab1_extraction_1** MRFIFGLLVSLVIVYTYYYEVQSVELRCPCTDGFRNPLFGLFLIGHDPPH

**G10c_146F.ab1_extraction_1** MRFIFGLLVSLVIVYTYYYEVQSVELRCPCTDGFRNPLFGLFLIGHDPPH

**G11c_extraction_1** MRFIFGLLVSLVIVYTYYYEVQSVELRCPCTDGFRNPLFGLFLIGHDPPH

**B1c_146F.ab1_extraction_1** MRFIFGLLVSLVIVYTYYYEVQSVELRCPCTDGFRNPLFGLFLIGHDPPH

**G5c_146F.ab1_extraction_1** MRFIFGLLVSLVIVYTYYYEVQSVELRCPCTDGFRNPLFGLFLIGHDPPH

**G3c_146F.ab1_extraction_1** MRFIFGLLVSLVIVYTYYYEVQSVELRCPCTDGFRNPLFGLFLIGHDPPH

105632893779#2.ab1__reversed__ MRFIFGLLVSLVIVYTYYYEVQSVELRCPCTDGFRNPLFGLFLIGHDPPH

A15_146F.ab1_extraction_1 MRFIFGLLVSLVIVYTYYYEVQSVELRCPCTDGFRNPLFGLFLIGHDPPH

**B2c_146F.ab1_extraction_1** MRFIFGLLVSLVIVYTYYYEVQSVELRCPCTDGFRNPLFGLFLIGHDPPH

481_146F.ab1_extraction_1 MRFIFGLLVSLVIVYTYYYEVQSVELRCPCTDGFRNPLFGLFLIGHDPPH

105599472009_146R.ab1__reverse MRFIFGLLVSLVIVYTYYYEVQSVELRCPCTDGFRNPLFGLFLIGHDPPH

105632424795#3.ab1__reversed__ MRFIFGLLVSLVIVYTYYYEVQSVELRCPCTDGFRNPLFGLFLIGHDPPH

1844_146R.ab1__reversed__extra MRFIFGLLVSLVIVYTYYYEVQSVELRCPCTDGFRNPLFGLFLIGHDPPH

6976_146R.ab1__reversed__extra MRFIFGLLVSLVIVYTYYYEVQSVELRCPCTDGFRNPLFGLFLIGHDPPH

2094_146R.ab1__reversed__extra MRFIFGLLVSLVIVYTYYYEVQSVELRCPCTDGFRNPLFGLFLIGHDPPH

105599212368_146R.ab1__reverse MRFIFGLLVSLVIVYTYYYEVQSVELRCPCTDGFRNPLFGLFLIGHDPPH

2968_146R.ab1__reversed__extra MRFIFGLLVSLVIVYTYYYEVQSVELRCPCTDGFRNPLFGLFLIGHDPPH

9140_146F.ab1_extraction_1 MRFIFGLLVSLVIVYTYYYEVQSVELRCPCTDGFRNPLFGLFLIGHDPPH

**C10a_146F.ab1_extraction_1** MRFIFGLLVSLVIVYTYYYEVQSVELRCPCTDGFRNPLFGLFLIGHDPPH

**A8c_146F.ab1_extraction_1** MRFIFGLLVSLVIVYTYYYEVQSVELRCPCTDGFRNPLFGLFLIGHDPPH

**C2b_146F.ab1_extraction_1** MRFIFGLLVSLVIVYTYYYEVQSVELRCPCTDGFRNPLFGLFLIGHDPPH

**C3c_146F.ab1_extraction_1** MRFIFGLLVSLVIVYTYYYEVQSVELRCPCTDGFRNPLFGLFLIGHDPPH

**C4c_146F.ab1_extraction_1** MRFIFGLLVSLVIVYTYYYEVQSVELRCPCTDGFRNPLFGLFLIGHDPPH

**C4b_146F.ab1_extraction_1** MRFIFGLLVSLVIVYTYYYEVQSVELRCPCTDGFRNPLFGLFLIGHDPPH

**C5b_146F.ab1_extraction_1** MRFIFGLLVSLVIVYTYYYEVQSVELRCPCTDGFRNPLFGLFLIGHDPPH

**C8c_146F.ab1_extraction_1** MRFIFGLLVSLVIVYTYYYEVQSVELRCPCTDGFRNPLFGLFLIGHDPPH

**F5c_extraction_1** MRFIFGLLVSLVIVYTYYYEVQSVELRCPCTDGFRNPLFGLFLIGHDPPH

**C9c_146F.ab1_extraction_1** MRFIFGLLVSLVIVYTYYYEVQSVELRCPCTDGFRNPLFGLFLIGHDPPH

**D8c_146F.ab1_extraction_1** MRFIFGLLVSLVIVYTYYYEVQSVELRCPCTDGFRNPLFGLFLIGHDPPH

**F6b_146F.ab1_extraction_1** MRFIFGLLVSLVIVYTYYYEVQSVELRCPCTDGFRNPLFGLFLIGHDPPH

**D5c_146F.ab1_extraction_1** MRFIFGLLVSLVIVYTYYYEVQSVELRCPCTDGFRNPLFGLFLIGHDPPH

**C11b_146F.ab1_extraction_1** MRFIFGLLVSLVIVYTYYYEVQSVELRCPCTDGFRNPLFGLFLIGHDPPH

**G1b_146F.ab1_extraction_1** MRFIFGLLVSLVIVYTYYYEVQSVELRCPCTDGFRNPLFGLFLIGHDPPH

**A3b_146F.ab1_extraction_1** MRFIFGLLVSLVIVYTYYYEVQSVELRCPCTDGFRNPLFGLFLIGHDPPH

**A12a_146F.ab1_extraction_1** MRFIFGLLVSLVIVYTYYYEVQSVELRCPCTDGFRNPLFGLFLIGHDPPH

105632069006#3.ab1__reversed__ MRFIFGLLVSLVIVYTYYYEVQSVELRCPCTDGFRNPLFGLFLIGHDPPH

105600529877_146R.ab1__reverse MRFIFGLLVSLVIVYTYYYEVQSVELRCPCTDGFRNPLFGLFLIGHDPPH

**E5a_146F.ab1_extraction_1** MRFIFGLLVSLVIVYTYYYEVQSVELRCPCTDGFRNPLFGLFLIGHDPPH

4708_146F.ab1_extraction_1 MRFIFGLLVSLVIVYTYYYEVQSVELRCPCTDGFRNPLFGLFLIGHDPPH

271_146F.ab1_extraction_1 MRFIFGLLVSLVIVYTYYYEVQSVELRCPCTDGFRNPLFGLFLIGHDLPH

105601769065#2.ab1_extraction_ MRFIFGLLVSLVIVYTYYYEVQSVELRCPCTDGFRNPLFGLFLIGHDPPH

105601042459#2.ab1__reversed__ MRFIFGLLVSLVIVYTYYYEVQSVELRCPCTDGFRNPLFGLFLIGHDPPH

4314_146R.ab1__reversed__extra MRFIFGLLVSLVIVYTYYYEVQSVELRCPCTDGFRNPLFGLFLIGHDPPH

105631985491_146F.ab1_extracti MRFIFGLLVSLVIVYTYYYEVQSVELRCPCTDGFRNPLFGLFLIGHDPPH

105599703329_146R.ab1__reverse MRFIFGLLVSLVIVYTYYYEVQSVELRCPCTDGFRNPLFGLFLIGHDPPH

4707_146R.ab1__reversed__extra MRFIFGLLVSLVIVYTYYYEVQSVELRCPCTDGFRNPLFGLFLIGHDPPH

8597_146R.ab1__reversed__extra MRFIFGLLVSLVIVYTYYYEVQSVELRCPCTDGFRNPLFGLFLIGHDPPH

8439_146R.ab1__reversed__extra MRFIFGLLVSLVIVYTYYYEVQSVELRCPCTDGFRNPLFGLFLIGHDPPH

6183_146R.ab1__reversed__extra MRFIFGLLVSLVIVYTYYYEVQSVELRCPCTDGFRNPLFGLFLIGHDPPH

8043_146R.ab1__reversed__extra MRFIFGLLVSLVIVYTYYYEVQSVELRCPCTDGFRNPLFGLFLIGHDPPH

**H9c_146F.ab1_extraction_1** MRFIFGLLVSLVIVYTYYYEVQSVELRCPCTDGFRNPLFGLFLIGHDPPH

**G12a_146F.ab1_extraction_1** MRFIFGLLVSLVIVYTYYYEVQSVELRCPCTDGFRNPLFGLFLIGHDPPH

**F2c_146F.ab1_extraction_1** MRFIFGLSVSLVIVYTYYYEVQSVELRCPCTDGFRNPLFGLFLIGHDPPH

**F12a_146F.ab1_extraction_1** MRFIFGLLVSLVIVYTCYYEVQSVELRCPCTDGFRNPLFGLFLIGHDPPH

9130_146R.ab1__reversed__extra MRFIFGLLVSLVIVYTYYYEVQSVELRCPCTDGFRNPLFGLFLIGHDPPH

******* ******** ****************************** **

**A6c_146F.ab1_extraction_1** PPVCERDQFFLKPPQGKTVCLGPEHHLSSWLKGQNSSLWHKVLVTKKGDE

**D03a_extraction__reversed__1** PPVCERDQFFLKPPQGKTVCLGPEHHLSSWLKGQNSSLWHKVLVTKKGDE

**C1a_146F.ab1_extraction_1** PPVCERDQFFLKPPQGKTVCLGPEHHLSSWLKGQNSSLWHKVLVTKKGDE

**A10a_extraction__reversed__1** PPVCERDQFFLKPPQGKTVCLGPEHHLSSWLKGQNSSLWHKVLVTKKGDE

**D11b_146F.ab1_extraction_1** PPVCERDQFFLKPPQGKTVCLGPEHHLSSWLKGQNSSLWHKVLVTKKGDE

**E11b_146F.ab1_extraction_1** PPVCERDQFFLKPPQGKTVCLGPEHHLSSWLKGQNSSLWHKVLVTKKGDE

**F5b_146F.ab1_extraction_1** PPVCERDQFFLKPPQGKTVCLGPEHHLSSWLKGQNSSLWHKVLVTKKGDE

**F6a_146F.ab1_extraction_1** PPVCERDQFFLKPPQGKTVCLGPEHHLSSWLKGQNSSLWHKVLVTKKGDE

**A5c_146F.ab1_extraction_1** PPVCERDQFFLKPPQGKTVCLGPEHHLSSWLKGQNSSLWHKVLVTKKGDE

**G10c_146F.ab1_extraction_1** PPVCGRDQFFLKPPQGKTVCLGPEHHLSSWLKGQNSSLWHKVLVTKKGDE

**G11c_extraction_1** PPVCGRDQFFLKPPQGKTVCLGPEHHLSSWLKGQNSSLWHKVLVTKKGDE

**B1c_146F.ab1_extraction_1** PPVCGRDQFFLKPPQGKTVCLGPEHHLSSWLKGQNSSLWHKVLVTKKGDE

**G5c_146F.ab1_extraction_1** PPVCGRDQFFLKPPQGKTVCLGPEHHLSSWLKGQNSSLWHKVLVTKKGDE

**G3c_146F.ab1_extraction_1** PPVCGRDQFFLKPPQGKTVCLGPEHHLSSWLKGQNSSLWHKVLVTKKGDE

105632893779#2.ab1__reversed__ PPVCGRDQFFLKPPQGKTVCLGPEHHLSSWLKGQNSSLWHKVLVTKKGDE

A15_146F.ab1_extraction_1 PPVCGRDQFFLKPPQGKTVCLGPEHHLSSWLKGQNSSLWHKVLVTKKGDE

**B2c_146F.ab1_extraction_1** PPVCGRDQFFLKPPQGKTVCLGPEHHLSSWLKGQNSSLWHKVLVTKKGDE

481_146F.ab1_extraction_1 PPVCGRDQFFLKPPQGKTVCLGPEHHLSSWLKGQNSSLWHKVLVTKKGDE

105599472009_146R.ab1__reverse PPVCGRDQFFLKPPQGKTVCLGPEHHLSSWLKGQNSSLWHKVLVTKKGDE

105632424795#3.ab1__reversed__ PPVCGRDQFFLKPPQGKTVCLGPEHHLSSWLKGQNSSLWHKVLVTKKGDE

1844_146R.ab1__reversed__extra PPVCGRDQFFLKPPQGKTVCLGPEHHLSSWLKGQNSSLWHKVLVTKKGDE

6976_146R.ab1__reversed__extra PPVCGRDQFFLKPPQGKTVCLGPEHHLSSWLKGQNSSLWHKVLVTKKGDE

2094_146R.ab1__reversed__extra PPVCGRDQFFLKPPQGKTVCLGPEHHLSSWLKGQNSSLWHKVLVTKKGDE

105599212368_146R.ab1__reverse PPVCGRDQFFLKPPQGKTVCLGPEHHLSSWLKGQNSSLWHKVLVTKKGDE

2968_146R.ab1__reversed__extra PPVCGRDQFFLKPPQGKTVCLGPEHHLSSWLKGQNSSLWHKVLVTKKGDE

9140_146F.ab1_extraction_1 PPVCGRDQFFLKPPQGKTVCLGPEHHLSSWLKGQNSSLWHKVLVTKKGDE

**C10a_146F.ab1_extraction_1** PPVCGRDQFFLKPPQGKTVCLGPEHHLSSWLKGQNSSLWHKVLVTKKGDE

**A8c_146F.ab1_extraction_1** PPVCGRDQFFLKPPQGKTVCLGPEHHLSSWLKGQNSSLWHKVLVTKKGDE

**C2b_146F.ab1_extraction_1** PPVCGRDQFFLKPPQGKTVCLGPEHHLSSWLKGQNSSLWHKVLVTKKGDE

**C3c_146F.ab1_extraction_1** PPVCGRDQFFLKPPQGKTVCLGPEHHLSSWLKGQNSSLWHKVLVTKKGDE

**C4c_146F.ab1_extraction_1** PPVCGRDQFFLKPPQGKTVCLGPEHHLSSWLKGQNSSLWHKVLVTKKGDE

**C4b_146F.ab1_extraction_1** PPVCGRDQFFLKPPQGKTVCLGPEHHLSSWLKGQNSSLWHKVLVTKKGDE

**C5b_146F.ab1_extraction_1** PPVCGRDQFFLKPPQGKTVCLGPEHHLSSWLKGQNSSLWHKVLVTKKGDE

**C8c_146F.ab1_extraction_1** PPVCGRDQFFLKPPQGKTVCLGPEHHLSSWLKGQNSSLWHKVLVTKKGDE

**F5c_extraction_1** PPVCGRDQFFLKPPQGKTVCLGPEHHLSSWLKGQNSSLWHKVLVTKKGDE

**C9c_146F.ab1_extraction_1** PPVCGRDQFFLKPPQGKTVCLGPEHHLSSWLKGQNSSLWHKVLVTKKGDE

**D8c_146F.ab1_extraction_1** PPVCGRDQFFLKPPQGKTVCLGPEHHLSSWLKGQNSSLWHKVLVTKKGDE

**F6b_146F.ab1_extraction_1** PPVCGRDQFFLKPPQGKTVCLGPEHHLSSWLKGQNSSLWHKVLVTKKGDE

**D5c_146F.ab1_extraction_1** PPVCGRDQFFLKPPQGKTVCLGPEHHLSSWLKGQNSSLWHKVLVTKKGDE

**C11b_146F.ab1_extraction_1** PPVCGRDQFFLKPPQGKTVCLGPEHHLSSWLKGQNSSLWHKVLVTKKGDE

**G1b_146F.ab1_extraction_1** PPVCGRDQFFLKPPQGKTVCLGPEHHLSSWLKGQNSSLWHKVLVTKKGDE

**A3b_146F.ab1_extraction_1** PPVCGRDQFFLKPPQGKTVCLGPEHHLSSWLKGQNSSLWHKVLVTKKGDE

**A12a_146F.ab1_extraction_1** PPVCGRDQFFLKPPQGKTVCLGPEHHLSSWLKGQNSSLWHKVLVTKKGDE

105632069006#3.ab1__reversed__ PPVCGRDQFFLKPPQGKTVCLGPEHHLSSWLKGQNSSLWHKVLVTKKGDE

105600529877_146R.ab1__reverse PPVCGRDQFFLKPPQGKTVCLGPEHHLSSWLKGQNSSLWHKVLVTKKGDE

**E5a_146F.ab1_extraction_1** PPVCGRDQFFLKSPQGKTVCLGPEHHLSSWLKGQNSSLWHKVLVTKKGDE

4708_146F.ab1_extraction_1 PPVCGRDQFFLKPPQGKTVCLGPEHHLSSWLKGQNSSLWHKVLVTKKGDE

271_146F.ab1_extraction_1 PPVCERDQFFLKPPQGKTVCLGPEHHLSSWLKGQNSSLWHKVLVTKKGDE

105601769065#2.ab1_extraction_ PPVCERDQFFLKPPQGKTVCLGPEHHLSSWLKGQNSSLWHKVLVTKKGDE

105601042459#2.ab1__reversed__ PPVCERDQFFLKPPQGKTVCLGPEHHLSSWLKGQNSSLWHKVLVTKKGDE

4314_146R.ab1__reversed__extra PPVCERDQFFLKPPQGKTVCLGPEHHLSSWLKGQNSSLWHKVLVTKKGDE

105631985491_146F.ab1_extracti PPVCERDQFFLKPPQGKTVCLGPEHHLSSWLKGQNSSLWHKVLVTKKGDE

105599703329_146R.ab1__reverse PPVCERDQFFLKPPQGKTVCLGPEHHLSSWLKGQNSSLWHKVLVTKKGDE

4707_146R.ab1__reversed__extra PPVCERDQFFLKPPQGKTVCLGPEHHLSSWLKGQNSSLWHKVLVTKKGDE

8597_146R.ab1__reversed__extra PPVCERDQFFLKPPQGKTVCLGPEHHLSSWLKGQNSSLWHKVLVTKKGDE

8439_146R.ab1__reversed__extra PPVCERDQFFLKPPQGKTVCLGPEHHLSSWLKGQNSSLWHKVLVTKKGDE

6183_146R.ab1__reversed__extra PPVCERDQFFLKPPQGKTVCLGPEHHLSSWLKGQNSSLWHKVLVTKKGDE

8043_146R.ab1__reversed__extra PPVCERDQFFLKPPQGKTVCLGPEHHLSSWLKGQNSSLWHKVLVTKKGDE

**H9c_146F.ab1_extraction_1** PPVCERDQFFLKPPQGKTVCLGPEHHLSSWLKGQNSSLWHKVLVTKKGDE

**G12a_146F.ab1_extraction_1** PPVCERDQFFLKPPQGKTVCLGPEHHLSSWLKGQNSSLWHKVLVTKKGDE

**F2c_146F.ab1_extraction_1** PPVCERDQFFLKPPQGKTVCLGPEHHLSSWLKGQNSSLWHKVLVTKKGDE

**F12a_146F.ab1_extraction_1** PPVCERDQFFLKPPQGKTVCLGPEHHLSSWVKGKNSSLWHKVLVTKKGDE

9130_146R.ab1__reversed__extra PPVCERDQFFLKPPQGKTVCLGPEHHLSSWVKGQNSSLWHKVLVTKKGDE

**** *******.*****************:**:****************

**A6c_146F.ab1_extraction_1** PQVQKRGEYPKNRARIIV

**D03a_extraction__reversed__1** PQVQKRGEYPKNRARIIV

**C1a_146F.ab1_extraction_1** PQVQKRGEYPKNRARIIV

**A10a_extraction__reversed__1** PQVQKRGEYPKNRARIIV

**D11b_146F.ab1_extraction_1** PQVQKRGEYPKNRARIIV

**E11b_146F.ab1_extraction_1** PQVQKRGEYPKNRARIIV

**F5b_146F.ab1_extraction_1** PQVQKRGEYPKNRARIIV

**F6a_146F.ab1_extraction_1** PQVQKRGEYPKNRARIIV

**A5c_146F.ab1_extraction_1** PQVQKRGEYPKNRARIIV

**G10c_146F.ab1_extraction_1** PQVQKRGEYPKNRARIIV

**G11c_extraction_1** PQVQKRGEYPKNRARIIV

**B1c_146F.ab1_extraction_1** PQVQKRGEYPKNRARIIV

**G5c_146F.ab1_extraction_1** PQVQKRGEYPKNRARIIV

**G3c_146F.ab1_extraction_1** PQVQKRGEYPKNRARIIV

105632893779#2.ab1__reversed__ PQVQKRGEYPKNRARIIV

A15_146F.ab1_extraction_1 PQVQKRGEYPKNRARIIV

**B2c_146F.ab1_extraction_1** PQVQKRGEYPKNRARIIV

481_146F.ab1_extraction_1 PQVQKRGEYPKNRARIIV

105599472009_146R.ab1__reverse PQVQKRGEYPKNRARIIV

105632424795#3.ab1__reversed__ PQVQKRGEYPKNRARIIV

1844_146R.ab1__reversed__extra PQVQKRGEYPKNRARIIV

6976_146R.ab1__reversed__extra PQVQKRGEYPKNRARIIV

2094_146R.ab1__reversed__extra PQVQKRGEYPKNRARIIV

105599212368_146R.ab1__reverse PQVQKRGEYPKNRARIIV

2968_146R.ab1__reversed__extra PQVQKRGEYPKNRARIIV

9140_146F.ab1_extraction_1 PQVQKRGEYPKNRARIIV

**C10a_146F.ab1_extraction_1** PQVQKRGEYPKNRARIIV

**A8c_146F.ab1_extraction_1** PQVQKRGEYPKNRARIIV

**C2b_146F.ab1_extraction_1** PQVQKRGEYPKNRARIIV

**C3c_146F.ab1_extraction_1** PQVQKRGEYPKNRARIIV

**C4c_146F.ab1_extraction_1** PQVQKRGEYPKNRARIIV

**C4b_146F.ab1_extraction_1** PQVQKRGEYPKNRARIIV

**C5b_146F.ab1_extraction_1** PQVQKRGEYPKNRARIIV

**C8c_146F.ab1_extraction_1** PQVQKRGEYPKNRARIIV

**F5c_extraction_1** PQVQKRGEYPKNRARIIV

**C9c_146F.ab1_extraction_1** PQVQKRGEYPKNRARIIV

**D8c_146F.ab1_extraction_1** PQVQKRGEYPKNRARIIV

**F6b_146F.ab1_extraction_1** PQVQKRGEYPKNRARIIV

**D5c_146F.ab1_extraction_1** PQVQKRGEYPKNRARIIV

**C11b_146F.ab1_extraction_1** PQVQKRGEYPKNRARIIV

**G1b_146F.ab1_extraction_1** PQVQKRGEYPKNRARIIV

**A3b_146F.ab1_extraction_1** PQVQKRGEYPKNRARIIV

**A12a_146F.ab1_extraction_1** PQVQKRGEYPKNRARIIV

105632069006#3.ab1__reversed__ PQVQKRGEYPKNRARIIV

105600529877_146R.ab1__reverse PQVQKRGEYPKNRARIIV

**E5a_146F.ab1_extraction_1** PQVQKRGEYPKNRARIIV

4708_146F.ab1_extraction_1 PQVQKRGEYPKNRARIIV

271_146F.ab1_extraction_1 PQVQKRGEYPKNRARIIV

105601769065#2.ab1_extraction_ PQVQKRGEYPKNRARIIV

105601042459#2.ab1__reversed__ PQVQKRGEYPKNRARIIV

4314_146R.ab1__reversed__extra PQVQKRGEYPKNRARIIV

105631985491_146F.ab1_extracti PQVQKRGEYPKNRARIIV

105599703329_146R.ab1__reverse PQVQKRGEYPKNRARIIV

4707_146R.ab1__reversed__extra PQVQKRGEYPKNRARIIV

8597_146R.ab1__reversed__extra PQVQKRGEYPKNRARIIV

8439_146R.ab1__reversed__extra PQVQKRGEYPKNRARIIV

6183_146R.ab1__reversed__extra PQVQKRGEYPKNRARIIV

8043_146R.ab1__reversed__extra PQVQKRGEYPKNRARIIV

**H9c_146F.ab1_extraction_1** PQVQKRGEYPKNRARIIV

**G12a_146F.ab1_extraction_1** PQVQKRGEYPKNRARIIV

**F2c_146F.ab1_extraction_1** PQVQKRGEYPKNRARIIV

**F12a_146F.ab1_extraction_1** PQVQKRGEYPRNRARIIV

9130_146R.ab1__reversed__extra PQVQKRGEYPRNRARIIV

**********:*******

**GT13**

4076_146F.ab1_extraction_1 MRFIFGLLIGLVIVYTYYYEVQSTELRCPCTGGLHDPLYGIFYAGRDPPR

B21_146F.ab1_extraction_1 MRFIFGLLIGLVIVYTYYYEVQSTELRCPCTGGLHDPLYGIFYAGRDPPR

105602433559_146F.ab1_extracti MRFIFGLLIGLVIVYTYYYEVQSTELRCPCTGGLHDPLYGIFYAGRDPPR

A18_146R.ab1__reversed__extrac MRFIFGLLIGLVIVYTYYYEVQSTELRCPCTGGLHDPLYGIFYAGRDPPR

105602642000_146R.ab1__reverse MRFIFGLLIGLVIVYTYYYEVQSTELRCPCTGGLHDPLYGIFYAGRDPPR

2052_146R.ab1__reversed__extra MRFIFGLLIGLVIVYTYYYEVQSTELRCPCTGGLHDPLYGIFYAGRDPPR

105632155530#3.ab1__reversed__ MRFIFGLLIGLVIVYTYYYEVQSTELRCPCTGGLHDPLYGIFYAGRDPPR

105631990142#2.ab1__reversed__ MRFIFGLLIGLVIVYTYYYEVQSTELRCPCTGGLHDPLYGIFYAGRDPPR

105600533130#2.ab1__reversed__ MRFIFGLLIGLVIVYTYYYEVQSTELRCPCTGGLHDPLYGIFYAGRDPPR

**E10c_146F.ab1_extraction_1** MRFIFGLLIGLVIVYTYYYEVQSTELRCPCTGGLHDPLYGIFYAGRDPPR

**A8b_146F.ab1_extraction_1** MRFIFGLLIGLVIVYTYYYEVQSTELRCPCTGGLHDPLYGIFYAGRDPPR

**A1b_extraction_1** MRFIFGLLIGLVIVYTYYYEVQSTELRCPCTGGLHDPLYGIFYAGRDPPR

**C8b_extraction_1** MRFIFGLLIGLVIVYTYYYEVQSTELRCPCTGGLHDPLYGIFYAGRDPPR

**D4b_146F.ab1_extraction_1** MRFIFGLLIGLVIVYTYYYEVQSTELRCPCTGGLHDPLYGIFYAGRDPPR

**C11c_146F.ab1_extraction_1** MRFIFGLLIGLVIVYTYYYEVQSTELRCPCTGGLHDPLYGIFYAGRDPPR

**C9a_146F.ab1_extraction_1** MRFIFGLLIGLVIVYTYYYEVQSTELRCPCTGGLHDPLYGIFYAGRDPPR

**E8c_146F.ab1_extraction_1** MRFIFGLLIGLVIVYTYYYEVQSTELRCPCTGGLHDPLYGIFYAGRDPPR

**E3b_146F.ab1_extraction_1** MRFIFGLLIGLVIVYTYYYEVQSTELRCPCTGGLHDPLYGIFYAGRDPPR

**H2c_146F.ab1_extraction_1** MRFIFGLLIGLVIVYTYYYEVQSTELRCPCTGGLHDPLYGIFYAGRDPPR

2129_146R.ab1__reversed__extra MRFIFGLLIGLVIVYTYYYEVQSTELRCPCTGGLHDPLYGIFYAGRDPPR

**F8c_146F.ab1_extraction_1** MRFIFGLLIGLVIVYTYYYEVQSTELRCPCTGGLHDPLYGIFYAGRDPPR

**F1b_146F.ab1_extraction_1** MRFIFGLLIGLVIVYTYYYEVQSTELRCPCTGGLHDPLYGIFYAGRDPPR

**F10b_146F.ab1_extraction_1** MRFIFGLLIGLVIVYTYYYEVQSTELRCPCTGGLHDPLYGIFYAGRDPPR

**G8c_146F.ab1_extraction_1** MRFIFGLLIGLVIVYTYYYEVQSTELRCPCTGGLHDPLYGIFYAGRDPPR

**G9a_146F.ab1_extraction_1** MRFIFGLLIGLVIVYTYYYEVQSTELRCPCTGGLHDPLYGIFYAGRDPPR

**B12c_146F.ab1_extraction_1** MRFIFGLLIGLVIVYTYYYEVQSTELRCPCTGGLHDPLYGIFYAGRDPPR

**B10b_146F.ab1_extraction_1** MRFIFGLLIGLVIVYTYYYEVQSTELRCPCTNGLHDPLYGIFYAGRDPPR

**G3b_146F.ab1_extraction_1** MRFIFGLLIGLVIVYTYYYEVQSTELRCPCTNGLHDPLYGIFYAGRDPPR

8204_146R.ab1__reversed__extra MRFIFGLLIGLVIVYTYYYEVQSTELRCPCTNGLHDPLYGIFYAGRDPPR

105631664431_146R.ab1__reverse MRFIFGLLIGLVIVYTYYYEVQSTELRCPCTNGLHDPLYGIFYAGRDPPR

*******************************.******************

4076_146F.ab1_extraction_1 PPGCEKDQYYLKPPKGKAVCLGPQHHLSIWLNGQNSSLWHRVLVTGKNGN

B21_146F.ab1_extraction_1 PPGCEKDQYYLKPPKGKAVCLGPQHHLSIWLNGQNSSLWHRVLVTGKNGN

105602433559_146F.ab1_extracti PPGCEKDQYYLKPPKGKAVCLGPQHHLSIWLNGQNSSLWHRVLVTGKNGN

A18_146R.ab1__reversed__extrac PPGCEKDQYYLKPPKGKAVCLGPQHHLSIWLNGQNSSLWHRVLVTGKNGN

105602642000_146R.ab1__reverse PPGCEKDQYYLKPPKGKAVCLGPQHHLSIWLNGQNSSLWHRVLVTGKNGN

2052_146R.ab1__reversed__extra PPGCEKDQYYLKPPKGKAVCLGPQHHLSIWLNGQNSSLWHRVLVTGKNGN

105632155530#3.ab1__reversed__ PPGCEKDQYYLKPPKGKAVCLGPQHHLSIWLNGQNSSLWHRVLVTGKNGN

105631990142#2.ab1__reversed__ PPGCEKDQYYLKPPKGKAVCLGPQHHLSIWLNGQNSSLWHRVLVTGKNGN

105600533130#2.ab1__reversed__ PPGCEKDQYYLKPPKGKAVCLGPQHHLSIWLNGQNSSLWHRVLVTGKNGN

**E10c_146F.ab1_extraction_1** PPGCEKDQYYLKPPKGKAVCLGPQHHLSIWLNGQNSSLWHRVLVTGKNGN

**A8b_146F.ab1_extraction_1** PPGCEKDQYYLKPPKGKAVCLGPQHHLSIWLNGQNSSLWHRVLVTGKNGN

**A1b_extraction_1** PPGCEKDQYYLKPPKGKAVCLGPQHHLSIWLNGQNSSLWHRVLVTGKNGN

**C8b_extraction_1** PPGCEKDQYYLKPPKGKAVCLGPQHHLSIWLNGQNSSLWHRVLVTGKNGN

**D4b_146F.ab1_extraction_1** PPGCEKDQYYLKPPKGKAVCLGPQHHLSIWLNGQNSSLWHRVLVTGKNGN

**C11c_146F.ab1_extraction_1** PPGCEKDQYYLKPPKGKAVCLGPQHHLSIWLNGQNSSLWHRVLVTGKNGN

**C9a_146F.ab1_extraction_1** PPGCEKDQYYLKPPKGKAVCLGPQHHLSIWLNGQNSSLWHRVLVTGKNGN

**E8c_146F.ab1_extraction_1** PPGCEKDQYYLKPPKGKAVCLGPQHHLSIWLNGQNSSLWHRVLVTGKNGN

**E3b_146F.ab1_extraction_1** PPGCEKDQYYLKPPKGKAVCLGPQHHLSIWLNGQNSSLWHRVLVTGKNGN

**H2c_146F.ab1_extraction_1** PPGCEKDQYYLKPPKGKAVCLGPQHHLSIWLNGQNSSLWHRVLVTGKNGN

2129_146R.ab1__reversed__extra PPGCEKDQYYLKPPKGKAVCLGPQHHLSIWLNGQNSSLWHRVLVTGKNGN

**F8c_146F.ab1_extraction_1** PPGCEKDQYYLKPPKGKAVCLGPQHHLSIWLNGQNSSLWHRVLVTGKNGN

**F1b_146F.ab1_extraction_1** PPGCEKDQYYLKPPKGKAVCLGPQHHLSIWLNGQNSSLWHRVLVTGKNGN

**F10b_146F.ab1_extraction_1** PPGCEKDQYYLKPPKGKAVCLGPQHHLSIWLNGQNSSLWHRVLVTGKNGN

**G8c_146F.ab1_extraction_1** PPGCEKDQYYLKPPKGKAVCLGPQHHLSIWLNGQNSSLWHRVLVTGKNGN

**G9a_146F.ab1_extraction_1** PPGCEKDQYYLKPPKGKAVCLGPQHHLSIWLNGQNSSLWHRVLVTGKNGN

**B12c_146F.ab1_extraction_1** PPGCEKDQYYLKPPKGKAVCLGPQHHLSIWLNGQNSSLWHKVLVTGKNGN

**B10b_146F.ab1_extraction_1** PPGCEKDQYYLKPPKGKAVCLGPHHHLSIWLNGQNSSLWHKVLVTGKNGN

**G3b_146F.ab1_extraction_1** PPGCEKDQYYLKPPKGKAVCLGPHHHLSIWLNGQNSSLWHKVLVTGKNGN

8204_146R.ab1__reversed__extra PPGCEKDQYYLKPPKGKAVCLGPHHHLSIWLNGQNSSLWHKVLVTGKNGN

105631664431_146R.ab1__reverse PPGCEKDQYYLKPPKGKAVCLGPHHHLSIWLNGQNSSLWHKVLVTGKNGN

***********************:****************:*********

4076_146F.ab1_extraction_1 GPHVTKKGDFPRGRKNIMI

B21_146F.ab1_extraction_1 GPHVTKKGDFPRGRKNIMI

105602433559_146F.ab1_extracti GPHVTKKGDFPRGRKNIMI

A18_146R.ab1__reversed__extrac GPHVTKKGDFPRGRKNIMI

105602642000_146R.ab1__reverse GPHVTKKGDFPRGRKNIMI

2052_146R.ab1__reversed__extra GPHVTKKGDFPRGRKNIMI

105632155530#3.ab1__reversed__ GPHVTKKGDFPRGRKNIMI

105631990142#2.ab1__reversed__ GPHVTKKGDFPRGRKNIMI

105600533130#2.ab1__reversed__ GPHVTKKGDFPRGRKNIMI

**E10c_146F.ab1_extraction_1** GPHVTKKGDFPRGRKNIMI

**A8b_146F.ab1_extraction_1** GPHVTKKGDFPRGRKNIMI

**A1b_extraction_1** GPHVTKKGDFPRGRKNIMI

**C8b_extraction_1** GPHVTKKGDFPRGRKNIMI

**D4b_146F.ab1_extraction_1** GPHVTKKGDFPRGRKNIMI

**C11c_146F.ab1_extraction_1** GPHVTKKGDFPRGRKNIMI

**C9a_146F.ab1_extraction_1** GPHVTKKGDFPRGRKNIMI

**E8c_146F.ab1_extraction_1** GPHVTKKGDFPRGRKNIMI

**E3b_146F.ab1_extraction_1** GPHVTKKGDFPRGRKNIMI

**H2c_146F.ab1_extraction_1** GPHVTKKGDFPRGRKNIMI

2129_146R.ab1__reversed__extra GPHVTKKGNFPRGRKNIMI

**F8c_146F.ab1_extraction_1** GPHVTKKGDFPRGRKNIMI

**F1b_146F.ab1_extraction_1** GPHVTKKGDFPRGRKNIMI

**F10b_146F.ab1_extraction_1** GPHVTKKGDFPRGRKNIMI

**G8c_146F.ab1_extraction_1** GPHVTKKGDFPRGRKNIMI

**G9a_146F.ab1_extraction_1** GPHVTKKGDFPRGRKNIMI

**B12c_146F.ab1_extraction_1** GPHVTKKGDFPRGRKNIMI

**B10b_146F.ab1_extraction_1** GPHVTKKGDFPRGRKNIMI

**G3b_146F.ab1_extraction_1** GPHVTKKGDFPRGRKNIMI

8204_146R.ab1__reversed__extra GPHVTKKGDFPRGRKNIMI

105631664431_146R.ab1__reverse GPHVTKKGDFPRGRKNIMI

********:**********

**GT14**

**E10b_146F.ab1_extraction_1** MRLIFGLLIFFMVAYMCYEVQSVELRCPCTHNVLNRPIGGVFWIGRDPPK

**H5a_146F.ab1_extraction_1** MRLIFGLLIFFMVAYMCYEVQSVELRCPCTHNVLNRPIGGVFWIGRDPPK

**G1c_146F.ab1_extraction_1** MRLIFGLLIFFMVAYMCYEVQSVELRCPCTHNVLNRPIGGVFWIGRDPPK

7523_146F.ab1_extraction_1 MRLIFGLLIFFMVAYMCYEVQSVELRCPCTHNVLNRPIGGVFWIGRDPPK

105597221963_146R.ab1__reverse MRLIFGLLIFFMVAYMCYEVQSVELRCPCTHNVLNRPIGGVFWIGRDPPK

105633395640_146R.ab1__reverse MRLIFGLLIFFMVAYMCYEVQSVELRCPCTHNVLNRPIGGVFWIGRDPPK

**************************************************

**E10b_146F.ab1_extraction_1** PPECDKPQHFLLTSQGKTVCLGPDHHITKWLDGQNSNSWYKVFITTNGNN

**H5a_146F.ab1_extraction_1** PPECDKPQHFLLTSQGKTVCLGPDHHITKWLDGQNSNSWYKVFITTNGNN

**G1c_146F.ab1_extraction_1** PPECDKPQHFLLTSQGKTVCLGPDHHITKWLDGQNSNSWYKVFITTNGNN

7523_146F.ab1_extraction_1 PPECDKPQHFLLTSQGKTVCLGPDHHITKWLDGQNSNSWYKVFITTNGNN

105597221963_146R.ab1__reverse PPECDKPQHFLLTSQGKTVCLGPDHHITKWLDGQNSNSWYKVFITTNGNN

105633395640_146R.ab1__reverse PPECDKPQHFLLTSQGKTVCLGPDHHITKWLDGQNSNSWYKVFITTNGNN

**************************************************

**E10b_146F.ab1_extraction_1** GPQIHKRAEHNKRPKWKL

**H5a_146F.ab1_extraction_1** GPQIHKRAEHNKRPKWKL

**G1c_146F.ab1_extraction_1** GPQIHKRAEHNKRPKWKL

7523_146F.ab1_extraction_1 GPQIHKRAEHNKRPKWKL

105597221963_146R.ab1__reverse GPQIHKRAEHNKRPKWKL

105633395640_146R.ab1__reverse GPQIHKRAEHNKRPKWKL

******************
