# Supplementary material for: The frequency of cytomegalovirus non-ELR UL146 genotypes in neonates with congenital CMV disease is comparable to strains in the background population
Source: BMC Infect Dis. 2021 Apr 26;21:386. doi: 10.1186/s12879-021-06076-w (PMC8077815; doi:10.1186/s12879-021-06076-w)
Supplement: Supplementary file 2 — Additional file 2. [file 12879_2021_6076_MOESM2_ESM.docx]

**Table S1. Observed intra-genotype variations among 198 amino acid sequences.**

| **Genotype** |  | **Intra-genotype difference** | | | | |  | **Frequencies** | | |
| --- | --- | --- | --- | --- | --- | --- | --- | --- | --- | --- |
|  |  |  |  |  |  |  |  |  |  |  |
|  |  | **Conservative mutation** |  | **Non-conservative mutation** |  | **Deletions** |  | **Case strains** |  | **Control strains** |
|  |  |  |  |  |  |  |  |  |  |  |
|  |  |  |  |  |  |  |  |  |  |  |
| GT1 |  |  |  | N43S |  |  |  | 0/4 |  | 1/9 |
|  |  | D65N |  |  |  |  |  | 2/4 |  | 3/9 |
|  |  |  |  |  |  | G71-G72 |  | 2/4 |  | 3/9 |
|  |  |  |  | P72S |  |  |  | 1/4 |  | 0/9 |
|  |  |  |  | P72H |  |  |  | 0/4 |  | 2/9 |
|  |  |  |  | Y89R |  |  |  | 0/4 |  | 2/9 |
|  |  |  |  | Y89H |  |  |  | 2/4 |  | 4/9 |
|  |  | N90D |  |  |  |  |  | 2/4 |  | 3/9 |
|  |  |  |  | T100M |  |  |  | 1/4 |  | 0/9 |
|  |  |  |  | G104E |  |  |  | 0/4 |  | 2/9 |
|  |  |  |  | R116G |  |  |  | 2/4 |  | 3/9 |
| GT2 |  | R102K |  |  |  |  |  | 0/3 |  | 1/9 |
| GT3 |  | D88N |  |  |  |  |  | 1/2 |  | 0/2 |
| GT4 |  |  |  | T99N |  |  |  | 1/2 |  | 0/2 |
| GT5 |  | Y77H |  |  |  |  |  | 1/3 |  | 0 |
| GT6 |  | None |  | None |  | None |  | 0 |  | 0/2 |
| GT7 |  | S7T |  |  |  |  |  | 1/6 |  | 0/8 |
|  |  | G46A |  |  |  |  |  | 0/6 |  | 1/8 |
|  |  | K92R |  |  |  |  |  | 2/6 |  | 2/8 |
|  |  | L94F |  |  |  |  |  | 0/6 |  | 2/8 |
|  |  | K96R |  |  |  |  |  | 1/6 |  | 0/8 |
|  |  |  |  | G102E |  |  |  | 1/6 |  | 0/8 |
|  |  | E106Q |  |  |  |  |  | 1/6 |  | 0/8 |
| GT8 |  | S94T |  |  |  |  |  | 1/12 |  | 0/3 |
| GT9 |  | A13V |  |  |  |  |  | 4/11 |  | 4/8 |
|  |  | M21V |  |  |  |  |  | 5/11 |  | 4/8 |
|  |  |  |  | Y34L |  |  |  | 0/11 |  | 1/8 |
|  |  | F39L |  |  |  |  |  | 4/11 |  | 2/8 |
|  |  |  |  | W41L |  |  |  | 4/11 |  | 2/8 |
|  |  | I42V |  |  |  |  |  | 1/11 |  | 0/8 |
|  |  |  |  | W60L |  |  |  | 4/11 |  | 4/8 |
|  |  |  |  | P62L |  |  |  | 1/11 |  | 0/8 |
|  |  |  |  | S83L |  |  |  | 1/11 |  | 0/8 |
|  |  | R93K |  |  |  |  |  | 2/11 |  | 2/8 |
|  |  |  |  | D98G |  |  |  | 4/11 |  | 3/8 |
|  |  |  |  | G99E |  |  |  | 1/11 |  | 0/8 |
|  |  | R105K |  |  |  |  |  | 4/11 |  | 2/8 |
| GT10 |  | None |  | None |  | None |  | 0/3 |  | 0/2 |
| GT11 |  |  |  |  |  | G100 |  | 2/4 |  | 0/3 |
| GT12 |  |  |  | L8S |  |  |  | 1/37 |  | 0/26 |
|  |  |  |  | Y17C |  |  |  | 1/37 |  | 0/26 |
|  |  |  |  | P48L |  |  |  | 0/37 |  | 1/26 |
|  |  |  |  | G55E |  |  |  | 13/37 |  | 12/26 |
|  |  |  |  | P63S |  |  |  | 1/37 |  | 0/26 |
|  |  | L81V |  |  |  |  |  | 1/37 |  | 1/26 |
|  |  | Q84K |  |  |  |  |  | 1/37 |  | 0/26 |
|  |  | K111R |  |  |  |  |  | 1/37 |  | 1/26 |
| GT13 |  |  |  | G32N |  |  |  | 2/19 |  | 2/11 |
|  |  | Q74H |  |  |  |  |  | 2/19 |  | 2/11 |
|  |  | R91K |  |  |  |  |  | 3/19 |  | 2/11 |
|  |  | D109N |  |  |  |  |  | 0/19 |  | 1/11 |
| GT14 |  | None |  | None |  | None |  | 0/3 |  | 0/3 |
